# How Isomer and Conformer Structures Impact Dissociation Dynamics of Alkane Radical Cations

**DOI:** 10.1021/acs.jpca.5c04290

**Published:** 2025-09-08

**Authors:** Madison K. Minvielle, Mikaela Aftel, Timothy Hill, Hugo A. López Peña, Katharine Moore Tibbetts

**Affiliations:** Department of Chemistry, 6889 Virginia Commonwealth University, Richmond, Virginia 23284, United States

## Abstract

Ionization of alkanes to form radical cations activates their otherwise unreactive C–H bonds, facilitating important chemical processes such as hydrocarbon cracking. This work investigates the radical cation dissociation dynamics of hexane (C_6_H_14_) structural isomers by using femtosecond time-resolved mass spectrometry and quantum chemical calculations. All five isomers exhibit competition between the yields of fragment ions arising from direct C–C bond cleavage or dissociative rearrangement with hydrogen migration on dynamical time scales of ∼50–300 fs, suggesting that hydrogen migration in the metastable cations operates on such short time scales. Additional isomer- and conformer-specific dynamics are observed. Preferential dissociation pathways in the branched isomers are found to arise from geometric relaxation to cation structures with one elongated C–C bond. Coherent vibrational excitation along this elongated C–C bond in 3-methylpentane and 2,3-dimethylbutane results in ion yield oscillations in the first ∼300–400 fs after ionization. Enhanced depletion of the molecular ion signal in *n*-hexane compared to that in the branched isomers is attributed to a strongly coupled excited state in the most populated conformer that can be accessed by a two-photon transition. Collectively, these results provide a foundational understanding of dissociation dynamics in alkane radical cations and how these dynamics are affected by specific isomer and conformer structures.

## Graphical abstract

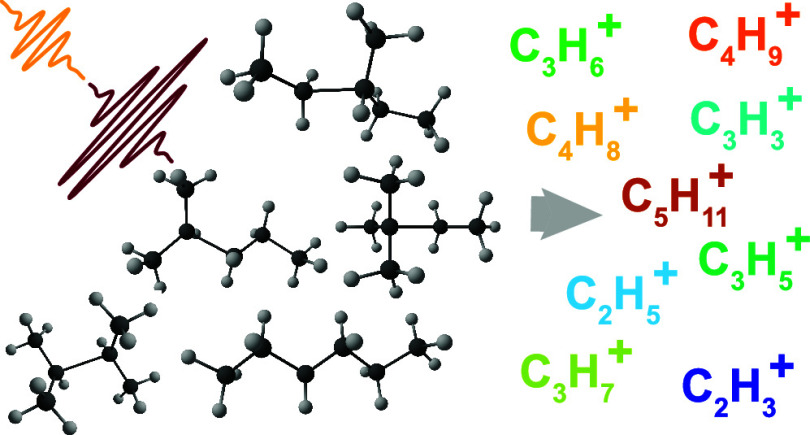

## 1 Introduction

Activation of C–H bonds in alkanes is important for many chemical processes including hydrocarbon cracking and the synthesis of pharmaceuticals. The formation of radical cations has long been recognized as a way to activate the otherwise unreactive C–H bonds in alkanes because alkane radical cations are prone to intramolecular hydrogen atom migration and rearrangement prior to dissociation.^1^ The propane radical cation, for example, preferentially undergoes hydrogen atom migration accompanied by C–C cleavage to form C_2_H_4_^+^ and CH_4_.^2–4^ This type of rearrangement also serves as a model reaction for C–H activation in photocatalysis reactions initiated by hydrogen atom abstraction.^5^

Mass spectrometry has been used for decades to study C–H activation and rearrangement reactions in alkane radical cations, with several studies documenting their intramolecular rearrangement and subsequent dissociation through isotopic labeling.^6–8^ For instance, the loss of neutral C_2_H_6_ to form C_4_H_8_^+^ from C_6_H_14_^+^ cations was found to involve distinct isomerization and hydrogen migration steps, depending on the initial isomer (*n*-hexane, 2-methylpentane, 3-methylpentane, or 2,3-dimethylbutane).^8^ Moreover, electron impact ionization studies^9^ have shown that fragment ions from C_6_H_14_ isomers produced by dissociative rearrangement reactions of the type

$$C_{6}{H_{14}}^{+}\to C_{6-m}{H_{14-(2m+2)}}^{+}+C_{m}H_{2m+2}\;(m=2,3)$$ 1

have lower appearance energies than ions produced by the corresponding direct bond cleavage reactions

$$C_{6}{H_{14}}^{+}\to C_{6-m}{H_{14-(2m+1)}}^{+}+C_{m}H_{2m+1}\;(m=2,3)$$ 2

A subsequent single-photon ionization study of hexane isomers with 10.49 eV VUV photons observed higher yields of fragment ions arising from rearrangement reactions described by eq 1 than those arising from eq 2, even at elevated temperatures.^10^ More recently, substantially lower calculated energy barriers to the reactions in eq 1 compared to other dissociation reactions were reported for the *n*-hexane cation.^11^

Despite the extensive reports of hydrogen migration and isomerization reactions in alkane radical cations, the time scales required for these rearrangements cannot be captured with conventional mass spectrometry methods that operate on microsecond time scales. The optical pump–probe technique of femtosecond time-resolved mass spectrometry (FTRMS) is uniquely suited to capturing the dynamics of intramolecular rearrangement and dissociation reactions in radical cations on femtosecond–picosecond time scales.^12,13^ FTRMS studies have identified the time scales associated with rearrangement reactions of radical cations including the nitro-nitrite rearrangement in nitromethane,^14^ the *aci*-hydrogen atom transfer in 2-nitrotoluene,^15^ and the hydrogen migration step of the McLafferty rearrangement in aliphatic ketones.^16^ Moreover, FTRMS measurements on *n*-butyl bromide found that the relative yields of C_3_H_*n*_^+^ (*n* = 7, 6, 5, 3) and C_2_H_*m*_^+^ (*m* = 5, 4, 3, 2) fragments evolved over subpicosecond time scales,^17^ suggesting that hydrogen migration dynamics in alkyl radical cations occur on such short time scales.

In addition to monitoring intramolecular rearrangements, FTRMS has revealed distinct dissociation dynamics in structural isomers of methylacetophenones,^18^ azobenzenes,^19^ and nitrotoluenes,^20,21^ despite the isomers having similar MS fragmentation patterns. This capability arises because the pump pulse induces strong-field ionization (SFI), which often yields a coherent vibrational excited state, or wave packet, whose dynamics can be monitored via ion yield oscillations as the pump–probe delay is varied.^12^ As a result of this coherent vibrational excitation, structural isomers were found to exhibit different phases^18,19^ or periods^20,21^ in their ion yield oscillations. Even dynamics of conformational isomers, or conformers, have been probed with FTRMS. Phase shifts in the fragment ion yield oscillations from dimethyl methylphosphonate (DMMP) were attributed to distinct electronic relaxation pathways from the D_3_ excited state that promoted dissociation into different fragments for the C_1_ and C_s_ conformers.^22^

This work investigates the dissociation dynamics of the five C_6_H_14_ isomers: *n*-hexane, 2-methylpentane, 3-methylpentane, 2,3-dimethylbutane, and 2,2-dimethylbutane. The competition between dissociative rearrangement and direct bond cleavage reactions is observed in the evolution of fragment ion yields on two distinct time scales of *T*_1_ ∼ 50–300 fs and *T*_2_ ∼ 1–5 ps in all isomers except 2,2-dimethylbutane. Additional isomer-specific ion yield dynamics are rationalized with density functional theory (DFT) and time-dependent DFT calculations of the structures and electronic excited-state energies. Moreover, the presence of three conformers in the *n*-hexane cation with distinct electronic structures explains the distinct ion yield dynamics observed in *n*-hexane compared to the branched isomers.

## 2 Methods

### 2.1 Experiment

The experimental setup has been described in detail in our previous work.^15,23,24^ A titanium-sapphire regenerative amplifier (Astrella, Coherent, Inc.) produced 2.2 mJ, 30 fs, and 800 nm pulses at 1 kHz that were used to pump an optical parametric amplifier (OPA). The signal (1427 nm) and idler (1800 nm) beams were used for the probe and pump pulses, respectively. The 1427 nm wavelength was frequency-doubled by using a beta-barium borate (BBO) crystal to produce the 713 nm probe wavelength. The 1800 nm pump and 713 nm probe beams were combined on a dichroic mirror and focused into the extraction region of a linear time-of-flight mass spectrometer (TOF-MS, Jordan TOF, Grass Valley, CA) using an *f* = 20 cm BaF_2_ lens to minimize negative GVD at the 1800 nm wavelength. The pump intensity was calibrated by measuring the saturation of O_2_^+^ (Supporting Information, Figure S1) and set to 7 × 10^13^ W cm^–2^. The probe intensity was calibrated by measuring the focal spot size, as in our previous work,^15^ and set to an intensity of approximately 1 × 10^13^ W cm^–2^. The instrument response function (IRF) was measured by the cross-correlation of the O_2_^+^ signal with the pump and probe beams to be about 35 fs fwhm (Figure S2).

The samples, *n*-hexane (99%, Millipore Sigma), 2-methylpentane (98%, TCI), 3-methyl-pentane (99%, Thermo Scientific), 2,3-dimethylbutane (98%, Millipore Sigma), and 2,2-dimethylbutane (98%, TCI), were used as received and introduced into the TOF-MS vacuum chamber by an effusive leak valve to maintain a working pressure of approximately 3 × 10^–7^ Torr. All sample vials were maintained at room temperature, with the exception that 2,2-dimethylbutane was set in an ice bath to minimize pressure fluctuations. Mass spectra were recorded for pump–probe delays from −500 to +7500 fs in 10 fs steps over the range of −200 to +800 and 50 fs otherwise. Mass spectra were recorded by averaging over 50,000 laser shots using a 1 GHz digital oscilloscope (WaveRunner 610Zi, Teledyne LeCroy). All reported ion yields were normalized to the area of the parent molecular ion signal (*m*/*z* 86), except for 2,2-dimethylbutane, which was normalized to the *m*/*z* 71 ion signal due to the low molecular ion signal.

### 2.2 Theory

Because alkanes can exist in multiple conformers with similar energies due to the presence of only sigma bonds, a two-step process was employed for identifying relevant conformer geometries for each C_6_H_14_ isomer. First, a sampling of ground-state conformational ensembles was generated using the conformer-rotamer ensemble sampling tool (CREST) version 2.12 ^25,26^ with the semiempirical extended tight-binding quantum chemistry package xTB version 6.6.1.^27^ CREST uses an iterative meta-dynamics genetic structure crossing (iMTD-GC) workflow with geometry optimization at the GFN2 level.^28^ This method was applied for the neutral parent species, with an energetic threshold for the generation of conformational ensembles of 2.0 kcal/mol. Second, the geometry of each identified conformer was optimized at the B3LYP/6-311+G­(d) level of theory^29–31^ using Gaussian 16.^32^ The B3LYP/6-311+G­(d) level of theory was chosen for geometry optimizations and frequency calculations due to its success in optimizing conformer geometries of alkyl amines in prior work.^33^ Frequency calculations were performed to confirm that the relaxed structures obtained were actual minima on the potential energy surface. The lowest energy conformers (three from *n*-hexane; two from 2-methylpentane; and one from 3-methylpentane, 2,3-dimethylbutane, and 2,2-dimethylbutane) were then optimized as cations. Geometric coordinates of each obtained structure are given in the Supporting Information, Tables S1–S16. Having these two sets of optimized neutral and cationic species, we performed calculations of vertical and adiabatic ionization energies. Electronic excited states for cations at both the neutral and cation geometries of each conformer were computed by time-dependent DFT (TDDFT) at the CAM-B3LYP/6–311+G­(d) level of theory. The CAM-B3LYP/6-311+G­(d) level of theory was chosen as it is long-range corrected and better suited for excited states compared to the level used for geometry optimization.^34^ Finally, the analysis under the frame of the quantum theory of atoms in molecules (QTAIM) was performed using Multiwfn version 3.8.^35^

## 3 Results and Discussion

### 3.1 Strong-Field Ionization (SFI) of Hexane Isomers

Figure 1 shows the mass spectra of *n*-hexane (red), 2-methylpentane (green), 3-methylpentane (blue), 2,3-dimethylbutane (magenta), and 2,2-dimethylbutane (orange), obtained with only the 1800 nm pump pulse at an intensity of 7 × 10^13^ W cm^–2^. This modest pump intensity was chosen to be below the threshold for the formation of multiply charged cations. The mass spectra of *n*-hexane and the methylpentanes match the spectra reported in our previous work^24^ under the same conditions: *n*-hexane shows little preference among C_2_–C_4_ fragments, whereas 2-methylpentane preferentially produces C_3_ and C_5_ fragments, and 3-methylpentane produces C_4_ fragments. Fragment selectivity is also observed in the dimethylbutane isomers: 2,3-dimethylbutane preferentially produces C_3_ fragments, whereas 2,2-dimethylbutane produces C_4_ and C_5_ fragments. 2,2-Dimethylbutane is unique among the structural isomers in that it produces almost no intact molecular ion signal. Our previous work rationalized the fragmentation selectivity in the methylpentanes by considering C–C bond cleavage reactions that produce a secondary carbocation.^24^ Here, we analyze the fragmentation patterns of all five isomers in terms of the computed geometric structures of the relaxed cations and experimental measurements of appearance energies and single-photon mass spectra from the literature.^9,10^

**Figure 1:**
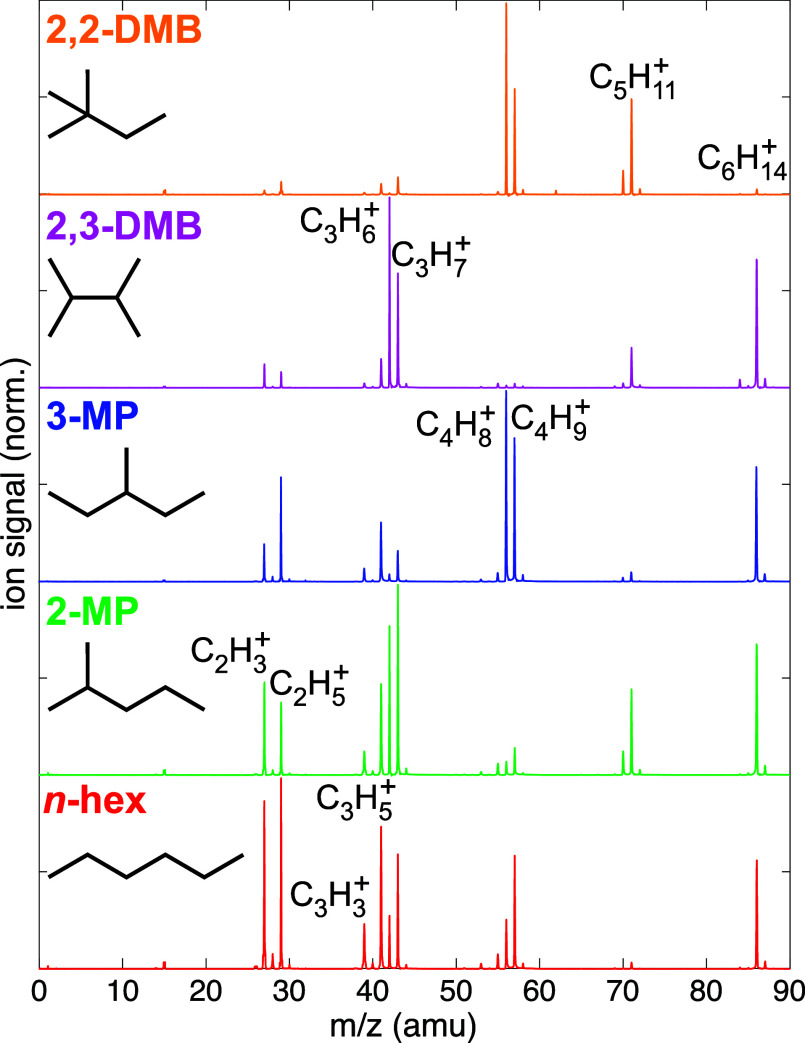
Pump-only mass spectra of *n*-hexane (*n*-hex, red), 2-methylpentane (2-MP, green), 3-methylpentane (3-MP, blue), 2,3-dimethylbutane (2,3-DMB, magenta), and 2,2-dimethylbutane (2,2-DMB, orange). Formulas of significant peaks are labeled.

Figure 2 shows the relaxed cation geometries obtained by DFT calculations for each hexane isomer. Both *n*-hexane and 2-methylpentane take on multiple conformers as neutral molecules (S_0_ geometry) at room temperature; the relative populations of each conformer at both the S_0_ and relaxed cation (D_0_) geometries at 25 °C are indicated in Figure 2. The three conformers of the *n*-hexane cation have similar C–C bond lengths but different bond angles. We note that the geometry of conformer 1 in Figure 2 is very close to the geometry for the *n*-hexane cation computed at the CAM-B3LYP/6-31++G* level in the literature.^11^ The lack of any significantly elongated C–C bonds in the *n*-hexane cation conformers can explain the lack of selectivity among C_2_H_*x*_^+^, C_3_H_*x*_^+^, or C_4_H_*x*_^+^ fragments in Figure 1. In contrast, all branched isomers have one significantly elongated bond as cations. Both conformers of 2-methylpentane have an elongated 1.96 Å C2–C3 bond, which explains the preferential fragmentation into C_3_H_*x*_^+^ fragments. Similarly, the preference for C_3_H_*x*_^+^ fragments in 2,3-dimethylbutane can be attributed to the elongation of the C2–C3 bond to 2.13 Å. Finally, elongation of the C2 to C3 bond in 3-methylpentane (1.97 Å) and 2,2-dimethylbutane (2.12 Å) explains the preferential fragmentation of both isomers to C_4_H_*x*_^+^ species.

**Figure 2:**
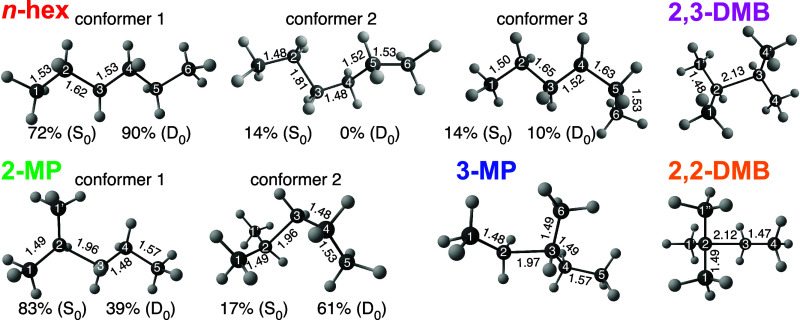
Geometric structures of cation conformers for *n*-hexane, 2-methylpentane, 3-methylpentane, 2,3-dimethylbutane, and 2,2-dimethylbutane computed at the B3LYP/6-311+G­(d) level of theory. Carbon atoms are labeled by number, and C–C bond lengths (in Å) are indicated for each structure. The relative populations of each conformer at 25 °C in *n*-hexane and 2-methylpentane for the S_0_ and D_0_ geometries are indicated.

At this point, it is worth investigating whether the elongated bonds within the branched hexane cations are actually bonds in the traditional sense. In order to explore this question, we used some descriptors emanating from the quantum theory of atoms in molecules (QTAIM).^36^ This theory, developed by Bader and collaborators, relies on quantum observables such as the electron density ρ­(**r**) and energy densities. We were able to locate critical points for the electron density corresponding to nuclear positions, called nuclear critical points (NCPs), and the so-called bond critical points (BCPs), which are critical points shared by chemically bonded atoms. We also found lines of locally maximum density, or bond paths, between the nuclei sharing a BCP. The collection of bond paths linking the nuclei of bonded atoms with the associated critical points is known as the molecular graph. Such molecular graphs for 2-MP^+^, 3-MP^+^, 2,3-DMB^+^, and 2,2-DMB^+^ are given in the Supporting Information, Figure S3, along with the coordinates for the NCPs and BCPs for each species in Tables S17–S20. It is important to emphasize that the identification of BCPs and bond paths between C2 and C3 for each cation species indicates the presence of some type of bonding interaction. To further classify the type of bonding, the following quantities at the BCP between C2 and C3 were calculated (Table 1): the electronic density (ρ_b_), the Laplacian of the electronic density (∇^2^ρ_b_), and the total electronic energy density (*H*_b_).

**Table 1:** QTAIM Bond Properties in Atomic Units (au) at the Bond Critical Point between C2 and C3 for the Relaxed Cations in Figure 2 (Except for *n*-Hexane)

cation	ρ_b_	∇^2^ρ_b_	*H*_b_
2-MP^+^	0.0813	–0.0034	–0.0293
3-MP^+^	0.0786	0.0012	–0.0274
2,3-DMB^+^	0.0584	0.0247	–0.0152
2,2-DMB^+^	0.0582	0.0255	–0.0151

It is generally accepted that ρ_b_ > 0.20 au indicates covalent bonding, and ρ_b_ < 0.10 au indicates a noncovalent interaction (van der Waals, for example). The Laplacian at the BCP is the sum of the three curvatures of the electron density at the critical point: the two perpendicular to the bond path, which are negative; and the third, lying along the bond path, is positive. The negative curvatures measure the extent to which the electron density is concentrated along the bond path, and the positive curvature measures the extent to which it is depleted in the interatomic region and concentrated near the atoms. In covalent bonding, the two negative curvatures are dominant and ∇^2^ρ_b_ < 0. In contrast, noncovalent interactions are characterized by a depletion of density in the interatomic region and ∇^2^ρ_b_ > 0. Energy densities are used to summarize the mechanics of the bonding interaction. The potential energy density, *V*(**r**), is the average effective potential field experienced by a single electron at point **r**, and it is always negative when evaluated at any point in space. *G*(**r**) is the gradient kinetic energy density and is always positive. Because *G*(**r**) and *V*(**r**) have opposite signs, Cremer and Kraka proposed the calculation of the total electronic energy density, *H*(**r**) = *G*(**r**) + *V*(**r**), at the BCP to characterize the bonding interaction: *H*_b_ = *G*_b_ + *V*_b_. *H*_b_ is negative for interactions with significant sharing of electrons, and its magnitude reflects the “covalence” of the interaction.^36,37^ Therefore, considering the values shown in Table 1 at the BCP between C2 and C3, we can classify the interactions as noncovalent according to the ρ_b_ values. Additionally, all the cations except 2-MP^+^ have ∇^2^ρ_b_ > 0, reinforcing the conclusion of having noncovalent interactions. On the other hand, the negative values for *H*_b_ for all the cations point toward interactions with some degree of electron sharing. Overall, the apparent contradiction between the different criteria suggests that the hexane cations represent borderline cases between noncovalent and covalent interactions. Resolving this contradiction deserves a more detailed study, but that is out of the scope of this work. Nevertheless, our current analysis gives insights into the existence of some sort of bonding interaction, which is reinforced by the fact that the parent ions are detected experimentally in significant yields in all branched isomers except 2,2-dimethylbutane.

The fragmentation patterns of the hexane isomers observed in Figure 1 can be further understood by considering their ionization and fragment appearance energies. Table 2 shows the vertical and adiabatic ionization energies for each isomer, calculated at the B3LYP/6-311+G­(d) level. For *n*-hexane and 2-methylpentane, the vertical and adiabatic ionization energies for the most populated neutral and cation conformers are reported (population analysis and vertical and adiabatic ionization energies for all conformers are given in the Supporting Information, Tables S21–S25). For comparison, the experimental vertical ionization energy^38^ and appearance energies for the relevant fragments^9^ in each isomer are shown. The calculated vertical ionization energies are generally 0.5 and 0.8 eV higher than the literature values.

**Table 2:** Calculated IE Values at the B3LYP/6-311+G­(d) Level with Experimental IE Values^38^ for Each Hexane Isomer and AE Values^9^ of Fragments from the Literature^a^

	*m*/*z*	*n*-hex	2-MP	3-MP	23-DMB	22-DMB
IE_vert_ calc.	86	10.49	10.52	10.84	10.49	10.56
IE_ad_ calc.	86	9.63	9.49	9.65	9.21	9.3
IE expt.	86	10.18	10.12	10.08	10.02	10.06
AE C_5_H_11_^+^	71	11.04	10.86	10.86	10.72	10.55
AE C_5_H_10_^+^	70	11.00	10.83	10.7	10.54	10.28
AE C_4_H_9_^+^	57	11.02	10.73	10.95		10.6
AE C_4_H_8_^+^	56	11.00	10.65	10.58		10.23
AE C_3_H_7_^+^	43	11.33	11.35		11.39	
AE C_3_H_6_^+^	42	11.00	10.91		10.69	

a All energies are reported in eV.

The significant yield of intact molecular ions in *n*-hexane, both methylpentanes, and 2,3-dimethylbutane in Figure 1 is consistent with the more than 0.5 eV gap between the experimental IE and lowest fragment AE values. In contrast, the small 0.16 eV gap between the IE and AE of C_4_H_8_^+^ in 2,2-dimethylbutane explains the extremely small yield of intact 2,2-dimethylbutane cations in Figure 1. This distinct behavior of 2,2-dimethylbutane is also consistent with previous measurements of single-photon ionization with 10.49 eV photons: mass spectra recorded at room temperature showed significantly higher yields of C_4_H_8_^+^ and C_5_H_11_^+^ than 2,2-dimethylbutane molecular ions, whereas the spectra of *n*-hexane, 3-methylpentane, and 2,3-dimethylbutane contained almost exclusively intact molecular ions.^10^ Nevertheless, the higher yields of fragment ions in all isomers obtained from SFI in Figure 1 compared to single-photon ionization suggest that a significant population of cations produced by SFI have internal energies in excess of the AE values for the respective fragments from Table 2, making them metastable to dissociation. To rationalize the production of electronically excited hexane cations during SFI, we turn to calculations of their excited-state energies in Section 3.2.

### 3.2 Electronic Structure and FTRMS Dynamics of C_6_H_14_^+^ Ions

Figure 3 shows the cation energy levels of each conformer from Figure 2 at both neutral (S_0_) and relaxed (D_0_) geometries, calculated at the CAM-B3LYP/6-311+G* level. Tabulated values of energies and oscillator strengths are given in the Supporting Information, Tables S26–S33. The relaxation energy, i.e., the energy difference between the cation under the S_0_ and D_0_ geometries, is in excess of 0.7 eV for all isomers and conformers, with the branched isomers having relaxation energies greater than 1 eV. Moreover, every isomer and conformer in the S_0_ geometry has multiple electronic excited states that lie within the energy of the pump and probe photons, illustrated by the dark and light red arrows in Figure 3. Hence, facile electronic excitation during or shortly after ionization combined with large relaxation energies is expected to result in cations with a large amount of excess energy that are metastable to dissociation. These results are consistent with the high yields of fragment ions observed in Figure 1. Previous studies on multiple classes of polyatomic molecules have shown that the presence of electronic excited states in the cation, with energies lower than the ionizing laser photon, tends to result in extensive fragmentation during SFI, while the absence of low-lying and strongly coupled excited states enhances the yield of intact molecular ions.^39–46^ In addition to this potential ionization into low-lying electronic excited states by the pump pulse, the energy of the 713 nm (1.74 eV) probe photon is capable of electronically exciting the initially formed cations in all isomers, which leads to the expectation that excitation at short pump–probe delays should enhance the yields of fragment ions.

**Figure 3:**
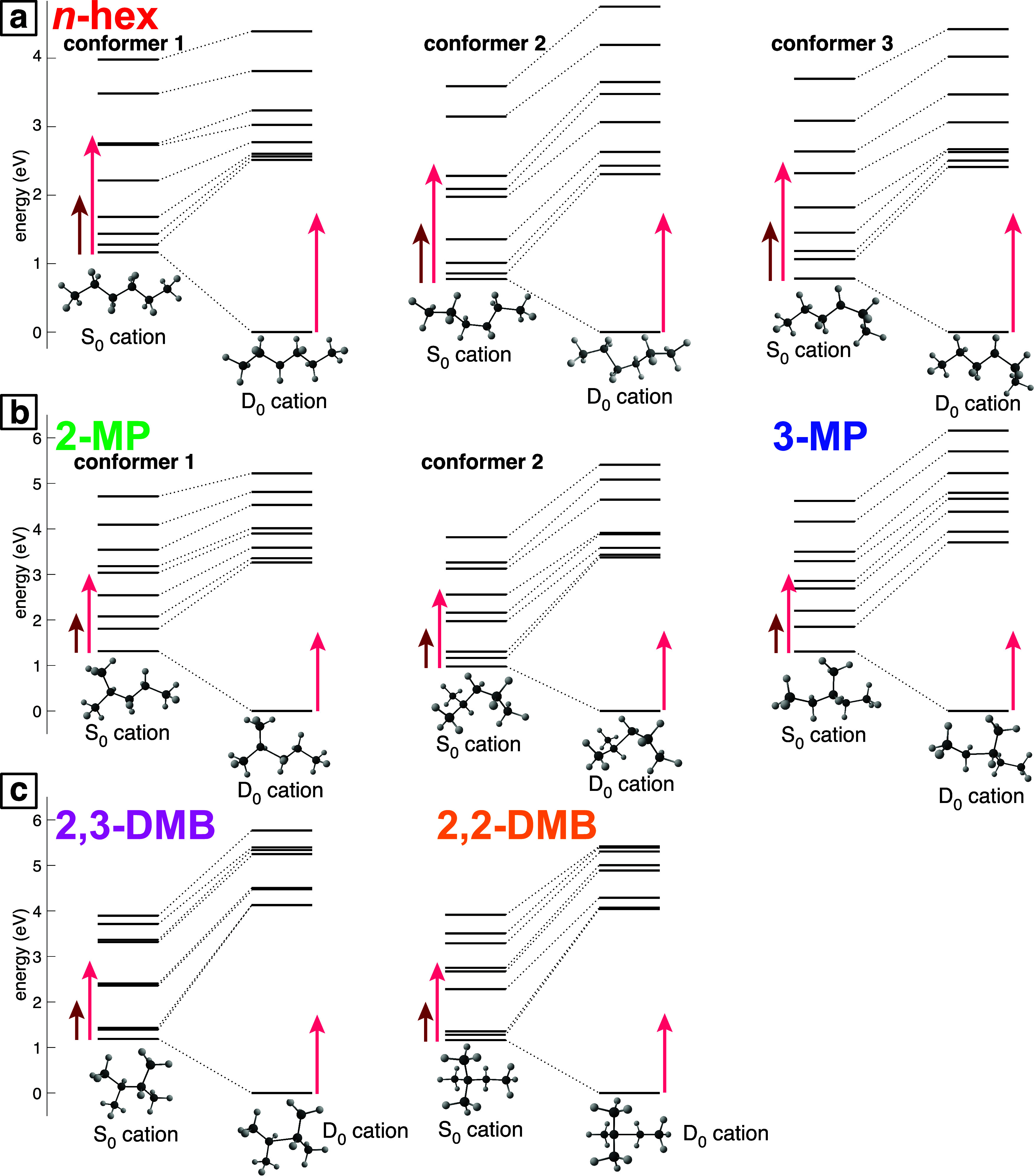
Electronic energy levels of the cation conformers for *n*-hexane (a), methylpentanes (b), and dimethylbutanes (c) under unrelaxed S_0_ and relaxed D_0_ geometries, calculated at the CAM-B3LYP/6-311+G* level of theory. Each diagram shows the S_0_ structure with its energy levels on the left and the D_0_ structure with its energy levels on the right. The dark and light red arrows denote the energies of the pump and probe photons, respectively.

It is evident from Figure 3 that geometric relaxation to relaxed D_0_ structures shifts the excited electronic states to significantly higher energies. For all isomer and conformer structures, the lowest-lying excited state is at a significantly higher energy than can be reached by the probe photon. The probe photon energy (1.74 eV) cannot bridge even half of the energy gaps to the strongly coupled excited states in the branched isomers (>3.9 eV, see Supporting Information), meaning that two-photon excitation is also not possible. In contrast, the dominant *n*-hexane conformer 1 (90% at the D_0_ geometry) has extremely strong coupling (*f* = 0.28) to an excited state 2.6 eV above the ground state, which could be reached by two-photon excitation with a 1.74 eV probe pulse. Because the relatively high intensity of the probe pulse (∼10^13^ W cm^–2^) is expected to enable two-photon excitation, enhanced *n*-hexane fragmentation compared to the branched isomers may be expected at long pump–probe delays.

Figure 4 shows the transient C_6_H_14_^+^ molecular ion signals for each isomer. Consistent with the computational results showing lower-lying excited states in the *n*-hexane conformers compared to the branched isomers, *n*-hexane undergoes the greatest depletion of the molecular ion signal at positive pump–probe delays, with the signal remaining depleted to 40% of its initial value even at +7500 fs. In contrast, 2,2-dimethylbutane barely undergoes any depletion at a positive pump–probe delay, although the low parent molecular ion yield results in a noisy signal that could obscure any dynamics. Both methylpentanes and 2,3-dimethylbutane undergo a weak transient depletion of the molecular ion signal immediately after zero delay (inset, Figure 4), followed by partial recovery of the ion signals at longer pump–probe delays. These dynamics are consistent with the electronic structures in Figure 3b,c as discussed above.

**Figure 4:**
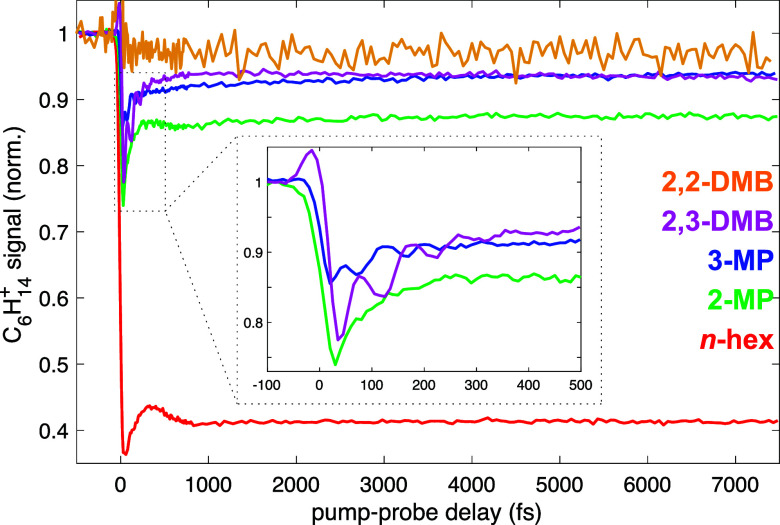
Transient C_6_H_14_^+^ ion signals for *n*-hexane (red), 2-methylpentane (green), 3-methylpentane (blue), 2,3-dimethylbutane (magenta), and 2,2-dimethylbutane (orange). The inset magnifies the dynamics of the methylpentanes and 2,3-dimethylbutane over the pump–probe delay range from −100 to +500 fs.

The molecular ion yields in 3-methylpentane and 2,3-dimethybutane exhibit oscillations at pump–probe delays below 500 fs (inset, Figure 4). Ion yield oscillations are commonly observed across many classes of organic molecules in FTRMS experiments due to coherent excitation of the molecular ion along one or more vibrational coordinates by the pump pulse.^13,15,18–22,47–52^ The oscillations in 3-methylpentane and 2,3-dimethylbutane may be further resolved after subtracting the exponential dynamics, as seen in Figure 5a,b (see Section 3.3 and the Supporting Information for details on fitting the exponential dynamics). Fast Fourier transform (FFT) of these oscillatory ion signals yields a broad feature that appears to contain a peak at 360 cm^–1^ with a shoulder at 470 cm^–1^ for 3-methylpentane (Figure 5c) and a well-resolved frequency of 340 cm^–1^ for 2,3-dimethylbutane (Figure 5d). Frequency analysis of the computed cation structures for each isomer (Supporting Information, Tables S34 and S35) reveals candidate modes at 327 and 471 cm^–1^ in the 3-methylpentane cation that exhibit displacement along the elongated C2–C3 bond (Figure 5e). The experimentally observed oscillations likely result from coherent excitation along one or both of these modes. One candidate mode was identified in the 2,3-dimethylbutane cation at 350 cm^–1^ that exhibits displacement along the elongated C2–C3 bond (Figure 5f). Such coherent vibrational excitation along the stretching coordinate of a bond that is significantly elongated upon ionization has also been observed in phosphonates, where elongation of the P=O bond upon ionization resulted in oscillatory ion signals of the molecular and fragment ions at the P=O frequency.^22,49,50^

**Figure 5:**
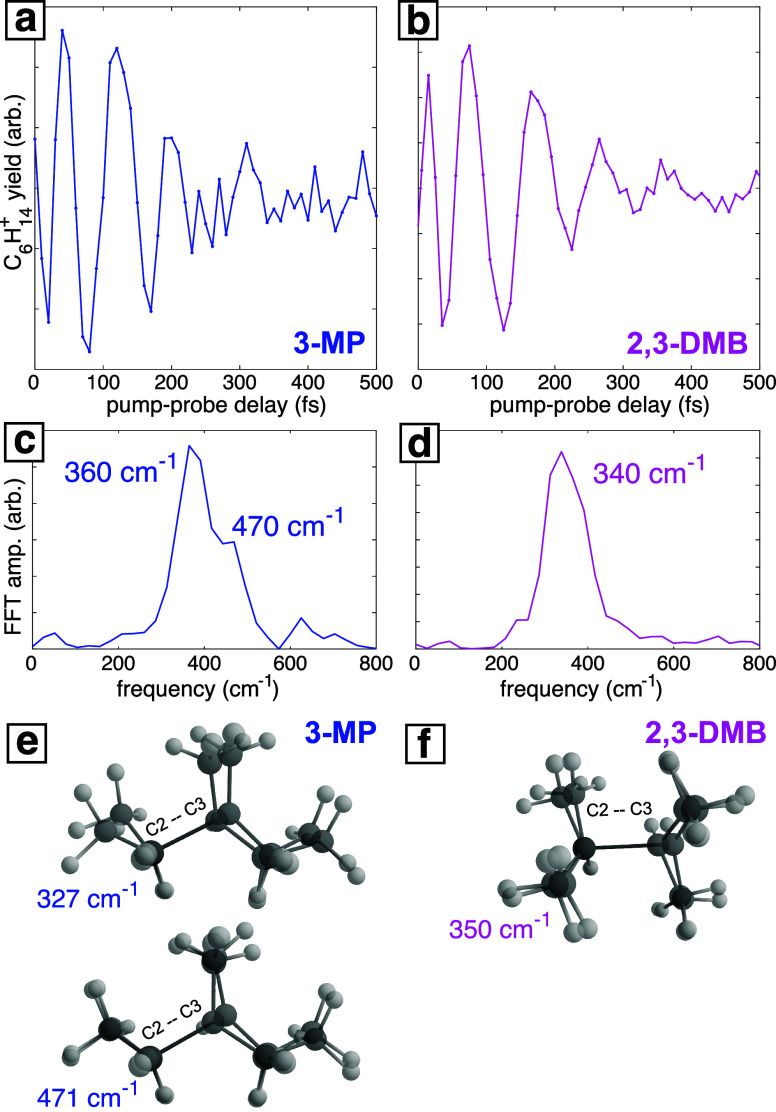
Analysis of oscillatory ion signals in 3-methylpentane (blue; (a), (c), (e)) and 2,3-dimethylbutane (magenta; (b), (d), (f)). Panels (a) and (b) show the C_6_H_14_^+^ ion yield signals after subtraction of exponential dynamics. Panels (c) and (d) show the fast Fourier transform (FFT) amplitudes of the signals in panels (a) and (b) with labeled frequencies. Panels (e) and (f) show the calculated structures and frequencies of vibrational modes in 3-methylpentane and 2,3-dimethylbutane cations with frequencies similar to those of the experimentally measured oscillations and displacement along the elongated C2–C3 bond.

Overall, the parent molecular ion dynamics of each isomer generally behave as would be expected from their electronic structures in Figure 3, with the observed dynamics arising from the population of molecules that were initially ionized into the D_0_ state by the pump pulse. Further insights into the dissociation dynamics of the electronically excited metastable cations, along with the fragment ions associated that are produced upon excitation of the vibrationally excited 3-methylpentane and 2,3-dimethylbutane cations, can be gained from examining the fragment ion dynamics discussed below in Section 3.3.

### 3.3 Fragment Ion Dynamics and Competitive Dissociation Pathways

The ion yield dynamics for the parent and major fragment ions in each hexane isomer over the pump–probe delay range of −100 to +500 fs are displayed in Figure 6 (the dynamics over the entire measured pump–probe delay range for each isomer are provided in the Supporting Information, Figures S4–S8). There are several trends in the fragment ion dynamics at short pump–probe delays that are common across all isomers. The larger fragments C_5_H_11_^+^, C_4_H_9_^+^, and C_4_H_8_^+^ tend to exhibit dynamics similar to those of the C_6_H_14_^+^ molecular ion, with depletion immediately after zero delay followed by a slow rise. The exception to this trend is the enhancement of the C_4_H_9_^+^ signal from 2,2-dimethylbutane immediately after zero delay. The smaller fragments C_2_H_3_^+^, C_2_H_5_^+^, C_3_H_3_^+^, and C_3_H_5_^+^ tend to exhibit the opposite dynamics: a sudden enhancement after zero delay followed by decay. These trends suggest that the initially produced electronically excited cations that would spontaneously dissociate into C_4_ or C_5_ fragments after ionization can be excited by the probe pulse, inducing dissociation instead into C_2_ or C_3_ fragments. In contrast to these general trends, the C_3_H_6_^+^ and C_3_H_7_^+^ fragment ions exhibit distinct dynamics in each isomer: both are initially depleted in *n*-hexane; C_3_H_6_^+^ is depleted and C_3_H_7_^+^ modestly enhanced in 2-methylpentane; and the C_3_H_6_^+^ and C_3_H_7_^+^ ions exhibit the opposite dynamics in 2,3-dimethylbutane, with C_3_H_6_^+^ depleted and C_3_H_7_^+^ enhanced.

**Figure 6:**
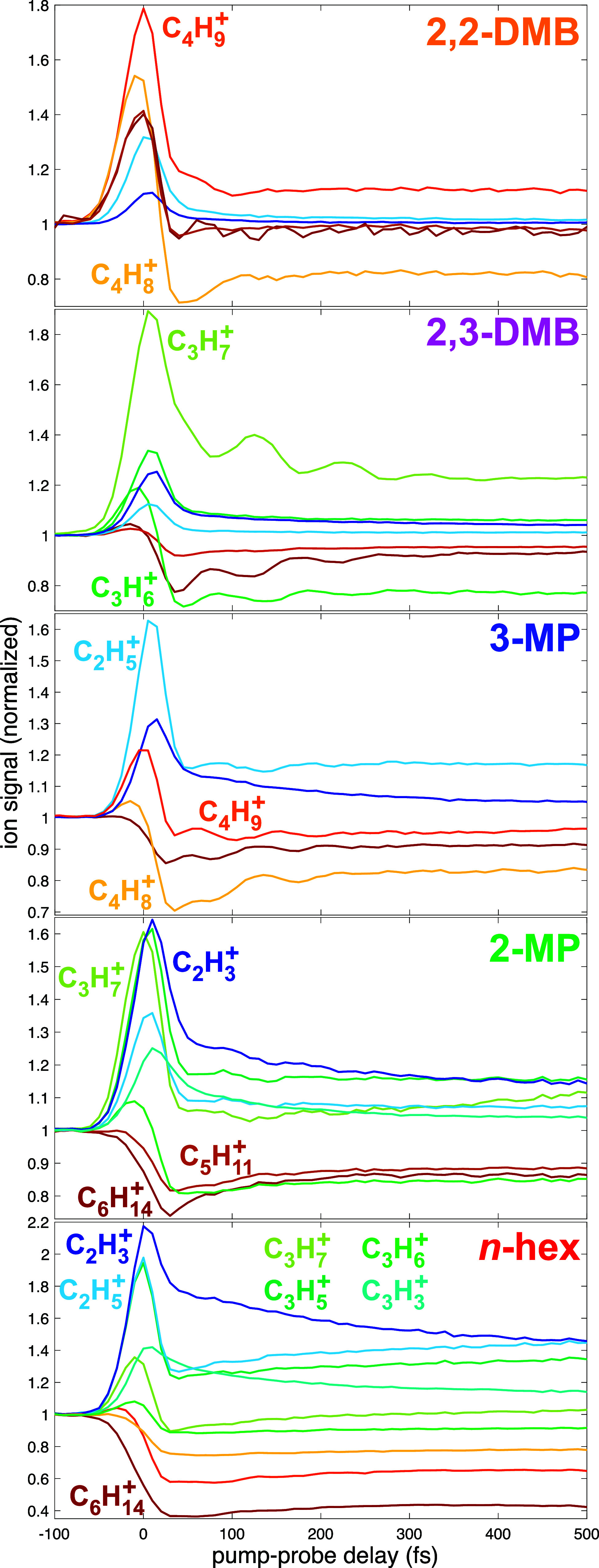
Transient dynamics of C_6_H_14_^+^ (dark red), C_5_H_11_^+^ (dark orange), C_4_H_9_^+^ (orange), C_4_H_8_^+^ (yellow), C_3_H_*x*_^+^ (*x* = 3, 5, 6, 7; shades of green), C_2_H_5_^+^ (light blue), and C_2_H_3_^+^ (dark blue) ions for each isomer over a delay range of −100 to +500 fs. All signals are normalized to 1.0 at a negative pump–probe delay.

The relative phases of the ion yield oscillations in fragments from 3-methylpentane and 2,3-dimethylbutane seen in Figure 6 are also of interest for identifying competing dissociation pathways. In 3-methylpentane, the oscillations of the molecular ion (dark red) and C_4_H_8_^+^ (yellow) can be seen to be roughly in-phase with each other and roughly out of phase with the C_4_H_9_^+^ signal (dark orange). Similarly, the oscillations in the molecular ion (dark red) and C_3_H_6_^+^ (green) signals from 2,3-dimethylbutane are roughly in phase with each other and out of phase with the C_3_H_7_^+^ (yellow-green) signal. The antiphase oscillations between pairs of fragment ions indicate that the dissociation pathways leading to fragmentation of 3-methylpentane into C_4_H_8_^+^ or C_4_H_9_^+^ and fragmentation of 2,3-dimethylbutane into C_3_H_6_^+^ and C_3_H_7_^+^ are in direct competition.^15,48,51^ Specifically, this result suggests that absorption of a photon from the probe pulse at a specific point along the potential energy surface can cause metastable 3-methylpentane ions that would spontaneously form C_4_H_8_^+^ to instead produce C_4_H_9_^+^. Similarly, metastable 2,3-dimethylbutane ions that would spontaneously form C_3_H_6_^+^ instead produce C_3_H_7_^+^. This competition between formation of C_4_H_8_^+^ or C_3_H_6_^+^, which involve hydrogen migration, and the direct C–C cleavage products C_4_H_9_^+^ or C_3_H_7_^+^ suggests that electronic excitation of metastable C_6_H_14_^+^ cations can selectively suppress spontaneous dissociation pathways involving migration of one hydrogen atom.

Further examination of the dynamics of the C_4_H_*x*_^+^ (*x* = 8, 9), C_3_H_*x*_^+^ (*x* = 3, 5, 6, 7), and C_2_H_*x*_^+^ (*x* = 3, 5) fragments for pump–probe delays up to +7500 fs reveals additional competing dissociation pathways among fragments with the same number of carbon atoms and different numbers of hydrogen atoms. These results suggest that competition between dissociation pathways involving hydrogen migration and direct bond cleavage is common across the hexane isomers. Figure 7 shows the transient ion signals of the C_4_H_*x*_^+^ (a), C_3_H_*x*_^+^ (b), and C_2_H_*x*_^+^ (c) fragments for each hexane isomer in which the fragments are produced in significant yields, as indicated on each panel.

**Figure 7:**
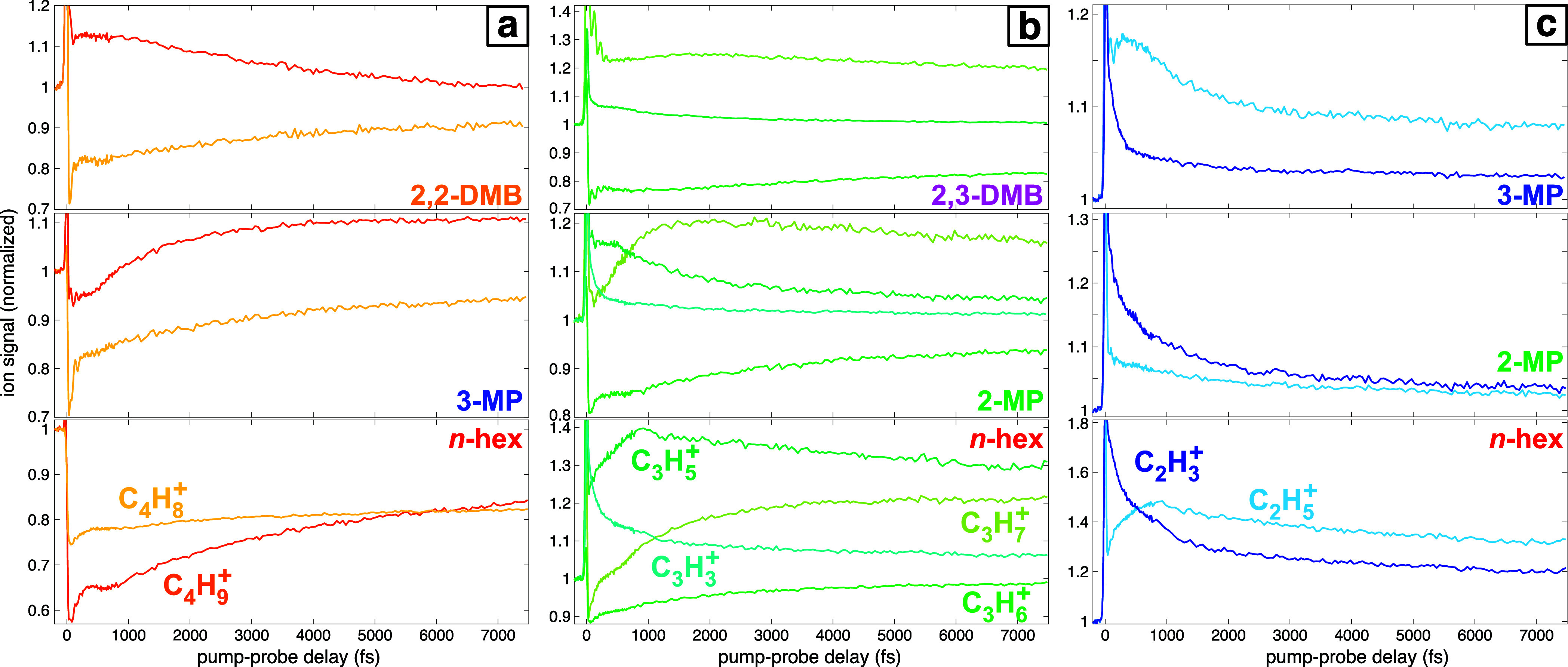
Transient ion signals of (a) C_4_H_*x*_^+^, (b), C_3_H_*x*_^+^, and (c) C_2_H_*x*_^+^ from the hexane isomers indicated on each panel. All ion signals are normalized to 1.0 at a negative pump–probe delay.

The C_4_H_9_^+^ and C_4_H_8_^+^ signals both exhibit initial depletion followed by a rise in *n*-hexane and 3-methylpentane (Figure 7a, bottom and middle). For *n*-hexane, both signals remain depleted below their initial yields at the end of the measurement window. For 3-methylpentane, the C_4_H_9_^+^ eventually rises to plateau at about 10% above its initial yield by +5000 fs. In contrast, the C_4_H_9_^+^ and C_4_H_8_^+^ fragments are directly in competition in 2,2-dimethylbutane (Figure 7a, top). This result is consistent with the lower appearance energy of C_4_H_8_^+^ than C_4_H_9_^+^ (Table 2) and suggests that the metastable 2,2-dimethylbutane cations that would spontaneously undergo hydrogen migration with C–C bond cleavage to produce C_4_H_8_^+^ and C_2_H_6_ instead undergo direct C–C bond cleavage to produce C_4_H_9_^+^ and C_2_H_5_ when excited by the probe pulse.

The C_3_H_*x*_^+^ fragments from *n*-hexane (Figure 7b, bottom) show a complex pattern of competition among pathways involving loss of 1, 2, or 4 hydrogen atoms that evolves over the measured pump–probe delay window. At short pump–probe delays below ∼800 fs, the decaying C_3_H_3_^+^ fragment exhibits the opposite dynamics to the rising C_3_H_5_^+^, C_3_H_6_^+^, and C_3_H_7_^+^ fragments. At longer pump–probe delays, the C_3_H_5_^+^ yield stops rising and instead decays, putting it in competition with the rising C_3_H_7_^+^ and C_3_H_6_^+^ yields. In 2-methylpentane (Figure 7b, middle), the initially depleted and rising C_3_H_6_^+^ signal is in direct competition with the initially enhanced and decaying C_3_H_5_^+^ and C_3_H_3_^+^ signals. The C_3_H_7_^+^ signal undergoes a slow rise in competition with C_3_H_5_^+^ and C_3_H_3_^+^ at short delays, followed by slow decay after +2000 fs. In 2,3-dimethylbutane (Figure 7b, top), the C_3_H_6_^+^ and C_3_H_7_^+^ fragments remain in competition through the 7500 fs measurement window. Collectively, the depletion of C_3_H_6_^+^ in all three isomers and enhancement of C_3_H_7_^+^ at long pump–probe delays is consistent with the lower appearance energies of C_3_H_6_^+^ relative to C_3_H_7_^+^ (Table 2). However, the further competition with pathways leading to C_3_H_5_^+^ and C_3_H_3_^+^ observed at short pump–probe delays points to more complex dissociation pathways involving migration of 2 or 4 hydrogen atoms occurring on subpicosecond time scales after ionization.

The dynamics of C_2_H_5_^+^ and C_2_H_3_^+^ in *n*-hexane (Figure 7c, bottom) show that these ions are in competition at <800 fs, while both signals decay in parallel at longer pump–probe delays. In contrast, both signals decay from their sudden enhancement after zero delay in 2-methylpentane (Figure 7c, middle), albeit at different rates. In 3-methylpentane (Figure 7c, top), the competition between C_2_H_5_^+^ and C_2_H_3_^+^ at <500 fs gives way to decay of both signals at longer pump–probe delays. Overall, the competition between C_2_H_5_^+^ and C_2_H_3_^+^ in *n*-hexane and 3-methylpentane at short pump–probe delays resembles the competition among C_3_H_5_^+^ and C_3_H_3_^+^ fragments seen in Figure 7b, suggesting similar short time scales for dissociation pathways to C_2_H_3_^+^ involving the loss of two hydrogen atoms.

To quantify the dynamical time scales seen in Figures 4 through 7, all transient ion signals were fit to exponential decay functions convoluted with the Gaussian instrument response function, following the literature method.^13^ Details on the fitting procedure, along with the resulting fitted dynamics for each ion signal and extracted time constants, are given in the Supporting Information, Figures S9–S14 and Tables S36–S41. Most ion signals required two exponential decay functions to fit the dynamics, with associated time constants *T*_1_ and *T*_2_. Tables 3 and 4 show these extracted time constants, along with the sign (positive or negative) of the associated amplitude coefficient, where ‘+’ indicates signal decay, and ‘–’ indicates signal rise.

**Table 3:** Time Constants for *T*_1_ (Femtoseconds) Extracted from Fitting Ion Dynamics^a^

ion (*m*/*z*)	*n*-hex	2-MP	3-MP	23-DMB	22-DMB
C_6_H_14_^+^ (86)		63 ± 7 (−)	60 ± 12 (−)	124 ± 9 (−)	
C_5_H_11_^+^ (71)	110 ± 40 (−)	81 ± 8 (−)		103 ± 13 (−)	
C_4_H_9_^+^ (57)	*x*		*x*		*x*
C_4_H_8_^+^ (56)	*x*		66 ± 7 (−)		*x*
C_3_H_7_^+^ (43)	*x*	800 ± 300 (−)		83 ± 15 (+)	
C_3_H_6_^+^ (42)	*x*	60 ± 20 (−)		60 ± 40 (−)	
C_3_H_5_^+^ (41)	260 ± 60 (−)	*x*		*x*	
C_3_H_3_^+^ (39)	125 ± 18 (+)	72 ± 5 (+)			
C_2_H_5_^+^ (29)	180 ± 30 (−)	*x*	*x*		
C_2_H_3_^+^ (27)	280 ± 80 (+)	50 ± 8 (+)	77 ± 8 (+)		

a Reported errors denote 95% confidence interval for coefficient fitting. The + and – signs indicate the sign of the associated amplitude coefficient. The ‘*x*’ denotes that a *T*_1_ coefficient could not be reliably determined; empty spaces denote that the ion signal was not subject to fitting.

**Table 4:** Time Constants for *T*_2_ (ps) Extracted from Fitting Ion Dynamics^a^

ion (*m*/*z*)	*n*-hex	2-MP	3-MP	23-DMB	22-DMB
C_6_H_14_^+^ (86)		2.1 ± 0.9 (−)	0.9 ± 0.2 (−)	*x*	
C_5_H_11_^+^ (71)	>5 (−)	>5 (−)		4 ± 2 (−)	
C_4_H_9_^+^ (57)	3.2 ± 0.3 (−)		3.2 ± 0.3 (−)		3.6 ± 1.4 (+)
C_4_H_8_^+^ (56)	1.9 ± 0.2 (−)		2.7 ± 0.3 (−)		1.8 ± 0.6 (−)
C_3_H_7_^+^ (43)	1.02 ± 0.09 (−)	>5 (+)		>5 (+)	
C_3_H_6_^+^ (42)	1.6 ± 0.1 (−)	2.9 ± 0.3 (−)		>5 (−)	
C_3_H_5_^+^ (41)	>5 (+)	1.7 ± 0.2 (+)		1.0 ± 0.1 (+)	
C_3_H_3_^+^ (39)	1.4 ± 0.2 (+)	1.4 ± 0.3 (+)			
C_2_H_5_^+^ (29)	4 ± 2 (+)	1.4 ± 0.1 (+)	1.7 ± 0.4 (+)		
C_2_H_3_^+^ (27)	1.7 ± 0.5 (+)	1.1 ± 0.1 (+)	1.1 ± 0.2 (+)		

a Reported errors denote 95% confidence interval for coefficient fitting. The + and – signs indicate the sign of the associated amplitude coefficient. The ‘*x*’ denotes that a *T*_2_ coefficient was not present; empty spaces denote that the ion signal was not subject to fitting.

The shorter *T*_1_ time constants are typically less than ∼300 fs, whereas the longer *T*_2_ time constants exceed 1 ps for most ions. For some ions, the reported *T*_2_ > 5 ps because longer time constants are not resolvable within our measurement window. It is notable that the range of *T*_1_ values for many ions that are formed by the loss of one or more hydrogen atoms (∼50–280 fs) are of similar magnitude to the ∼130–190 fs time constant reported for the hydrogen migration step of the McLafferty rearrangement in aliphatic ketones^16^ and the ∼50–330 fs time scales observed in hydrocarbon fragment ions from *n*-butyl bromide.^17^ This concurrence raises the possibility that the short *T*_1_ time scales obtained for ions such as C_4_H_8_^+^, C_3_H_*x*_^+^ (*x* = 6, 5, 3), and C_2_H_3_^+^ may be associated with a hydrogen migration that occurs before or simultaneously with C–C bond cleavage. The longer *T*_2_ time constant could be associated with an initial or sequential bond cleavage step. However, we note that further computational work, including molecular dynamics simulations, would be needed to support the assignment of any experimental time constant to a specific reaction process.

Even without such computations, visualization of the complex ion yield dynamics from the large numbers of fragments observed in the hexane isomers can be aided by calculating the Pearson correlation coefficient for each pair of ions over a specific range of pump–probe delays.^13,52^ The Pearson correlation coefficient is given by the equation

$$r_{ij}=\frac{N\left(\overset{N}{\underset{k=1}{\sum }}Y_{ik}Y_{jk}\right)-\left(\overset{N}{\underset{k=1}{\sum }}Y_{ik}\right)\left(\overset{N}{\underset{k=1}{\sum }}Y_{jk}\right)}{\sqrt{\left[N\overset{N}{\underset{k=1}{\sum }}Y_{ik}^{2}-{\left(\overset{N}{\underset{k=1}{\sum }}Y_{ik}\right)}^{2}\right]\left[N\overset{N}{\underset{k=1}{\sum }}Y_{jk}^{2}-{\left(\overset{N}{\underset{k=1}{\sum }}Y_{jk}\right)}^{2}\right]}}$$ 3

where *N* is the number pump–probe delays, and *Y*_*ik*_ and *Y*_*jk*_ denote the yields of the *i*th and *j*th ions at the *k*th time delay, respectively. Plotting the coefficients in a correlation diagram allows for direct visualization of the correlations between all pairs of relevant ions over a given pump–probe delay range.^13,52^ Because most ion dynamics from the C_6_H_14_ isomers were fit to two exponential decays, in some cases with opposite amplitudes, the Pearson coefficients were calculated separately here over the delay ranges of 50–800 fs and 800–7500 fs.

Figure 8 shows the correlation diagrams for each isomer, with the coefficients for the 50–800 fs delay range plotted in the top left and for the 800–7500 fs delay range plotted in the bottom right of each diagram. These diagrams allow for rapid discrimination between pairs of fragments with similar dynamics, which exhibit positive correlations, from pairs of competing fragments, which exhibit negative correlations. It is evident from Figure 8 that the correlation plots can change dramatically between short (<800 fs) and long pump–probe delays, as seen in the different patterns in the top left compared to the bottom right of each plot. Correlations between some pairs of ions in each isomer change from positive (red) to negative (blue) or vice versa when going from short to long pump–probe delays. For instance, *m*/*z* 29 in *n*-hexane is positively correlated with ions at *m*/*z* 41 or higher at short times, but the correlation turns negative at long times. The opposite change in correlations from negative to positive at long times is seen for the *m*/*z* 43 ion compared to the *m*/*z* 56 or higher ions in 2-methylpentane.

**Figure 8:**
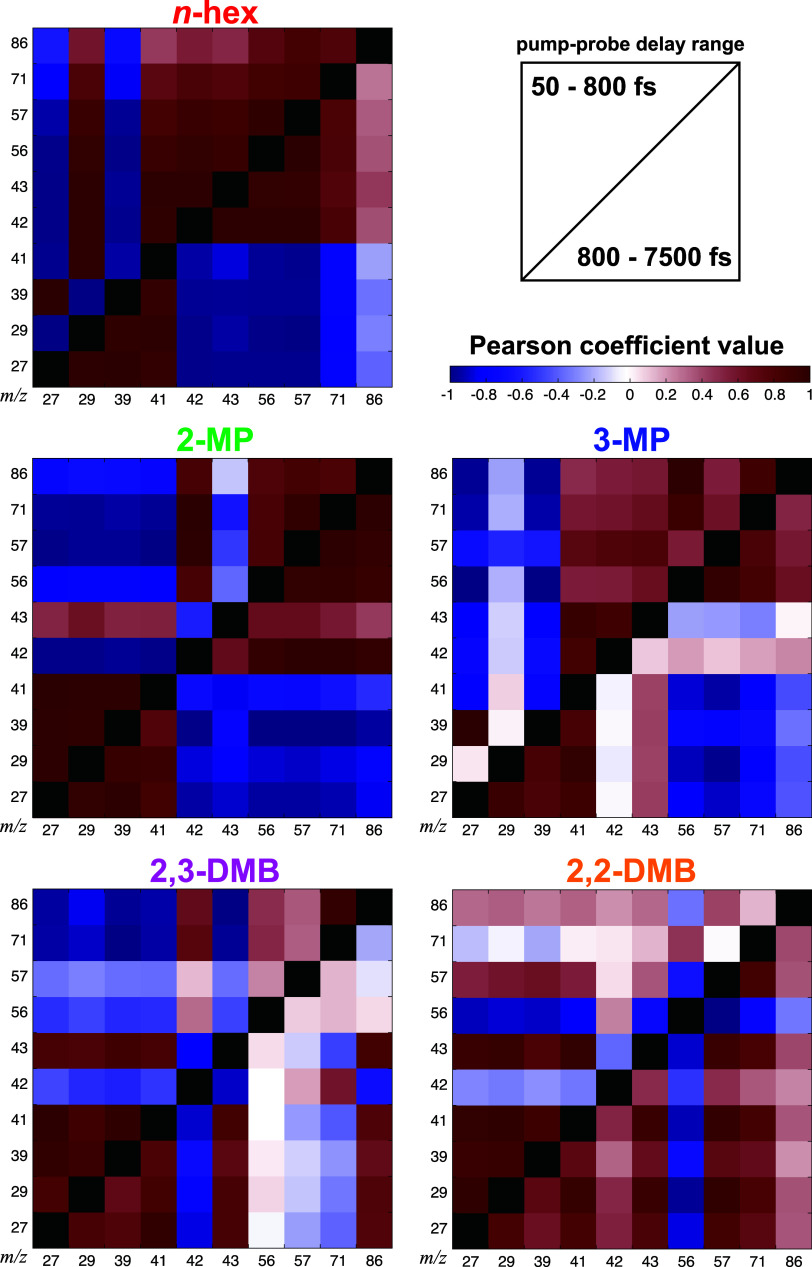
Pearson coefficients calculated using eq 3 for each C_6_H_14_ isomer. Positive correlations are in red, and negative correlations are in blue; the black squares along the diagonal represent the same ion on the ordinate and abscissa axis. As indicated in the image at the upper right of the figure, the upper left half of each plot shows the coefficients calculated for the pump–probe delay range of 50–800 fs, and the bottom right of each plot shows the coefficients calculated over the range of 800–7500 fs.

The correlation diagrams also clearly show the competition between many fragments with the same number of carbons but different numbers of hydrogens, particularly at short time delays. For instance, C_4_H_9_^+^ (*m*/*z* 57) and C_4_H_8_^+^ (*m*/*z* 56) are anticorrelated in 2,2-dimethylbutane, C_3_H_7_^+^ (*m*/*z* 43) and C_3_H_6_^+^ (*m*/*z* 42) are anticorrelated in 2-methylpentane and 2,3-dimethylbutane, and C_2_H_5_^+^ (*m*/*z* 29) and C_2_H_3_^+^ (*m*/*z* 27) are anticorrelated in *n*-hexane. Although these anticorrelations persist into the long pump–probe delay window for both dimethylbutanes, the prevalence of the anticorrelations in the short pump–probe delay window further supports the conjecture, based on the extracted time constants, that hydrogen migration in the hexane cations likely occurs on subpicosecond time scales. We note that the alkyl fragment migrations and rearrangements observed in previous MS studies of alkane radical cations (where ions were detected microseconds after ionization)^6–8^ likely occur on the several-picosecond *T*_2_ or even longer time scales due to their larger masses.

To summarize the findings on competing dissociation pathways and their dynamical time scales, Figure 9 depicts major fragment ions observed from each isomer, classified by the number of hydrogen atoms lost (“direct” indicates C–C bond cleavage without hydrogen loss). The light red arrows denote excitation pathways by the probe pulse between competing fragment ions. The line type of each arrow denotes the pump–probe delay range over which the competition between fragment ions is observed: <800 fs (dotted); >800 fs (dashed), or all delays measured (solid). The pairs of competing fragment ions and the time scales are primarily assigned based on negative Pearson coefficients in Figure 8, with two exceptions in 3-methylpentane. First, competition between C_2_H_5_^+^ and C_2_H_3_^+^ is evident from the gradually increasing C_2_H_5_^+^ yield compared to the decreasing C_2_H_3_^+^ yield over delays of up to 500 fs. Second, the competition between C_4_H_8_^+^ and C_4_H_9_^+^ at short delays is assigned based on the rough inverse-phase relationship between the oscillations in these two ion yields. Both of these features are visible in Figure 6.

**Figure 9:**
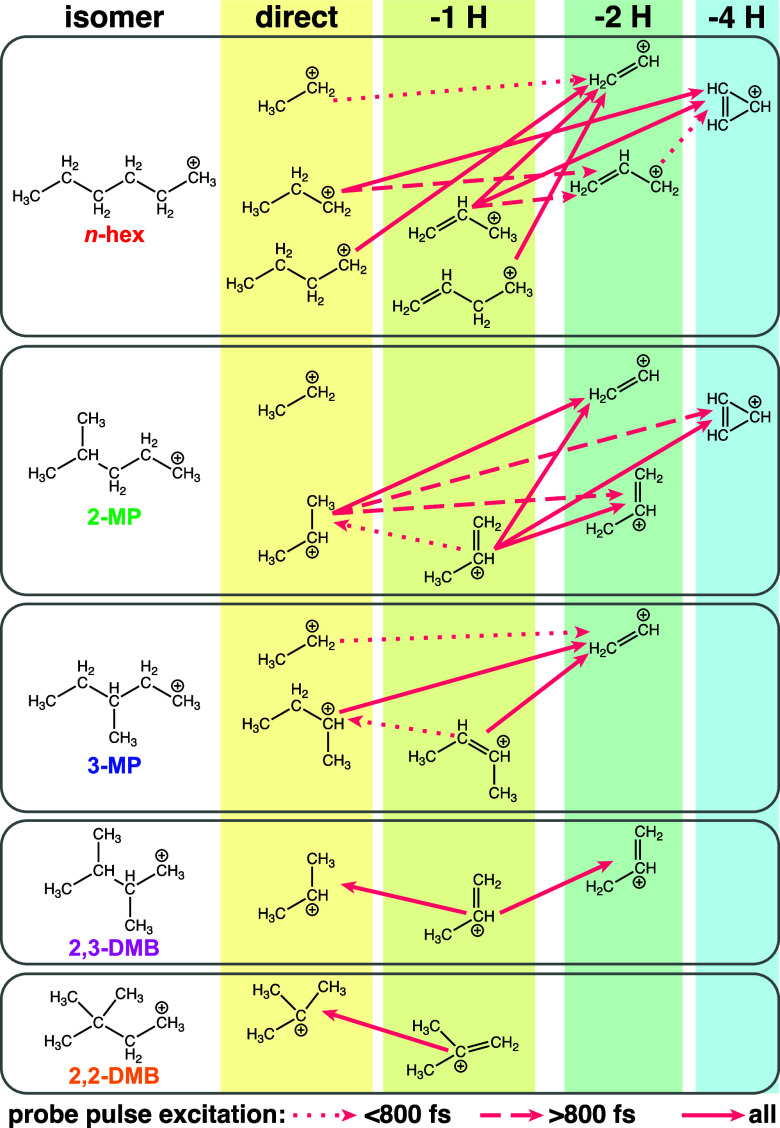
Schematic depiction of competing dissociation pathways from each hexane isomer. Fragment ions are classified according to the number of hydrogen atoms lost: 0 (“direct”, yellow), 1 (“–1 H”, yellow-green), 2 (“–2 H”, green), or 4 (“–4 H”, cyan). The light red arrows denote competing fragment ions, wherein excitation by the probe pulse depletes the initial fragment and enhances the final fragment. The line type denotes the time scale over which competition is observed: <800 fs (dotted); >800 fs (dashed), or all times (solid).

Comparison of the competing dissociation pathways across the hexane isomers reveals several patterns. First, C_3_H_6_^+^ or C_4_H_8_^+^ fragments (both formed by loss of one hydrogen) are in competition with the corresponding direct dissociation fragments (C_3_H_7_^+^ or C_4_H_9_^+^) in all branched isomers. However, this competition only holds for short pump–probe delays in the methylpentanes, whereas the competition persists over the entire measured delay range in the dimethylbutanes. Second, the yield of the C_2_H_3_^+^ ion, resulting from the loss of two hydrogen atoms, is enhanced upon electronic excitation of the precursor cation in both *n*-hexane and the methylpentanes, but enhancement at the expense of the direct dissociation product C_2_H_5_^+^ is only observed at short pump–probe delays. Finally, the observed competition between multiple pairs of fragments with different numbers of hydrogen losses in all isomers (except 2,2-dimethylbutane) suggests that multiple hydrogen migration pathways, operating on different time scales, generally operate in alkane radical cations.

Several common patterns emerge when comparing the results from this work to previous studies of radical cation dynamics with FTRMS. First, both the parent and fragment ions produced from each hexane isomer exhibited distinct dynamics, with different extracted decay times and competition patterns (Tables 3 and 4 and Figures 6–9). This finding is consistent with previous reports that found that radical cations from structural isomers exhibit distinct dissociation dynamics reflected in ion yield oscillations.^18–21^ Second, the observation of short (60 – 300 fs) time constants in many fragments that are formed by hydrogen migration is consistent with fast hydrogen migration times reported for the McLafferty rearrangement (130–190 fs) in alkyl ketone cations^16^ and aci-hydrogen transfer (∼20 fs) in the 2-nitrotoluene cation.^15^ Finally, we observe generally similar dynamical time constants in the fragment ions from hexane isomers to those reported from the substituted linear alkanes *n*-butyl bromide^17^ and *n*-pentyl nitrite.^53^ The *T*_2_ ∼ 3.2–3.6 ps in C_4_H_9_^+^ ions is close to the 4 ps time constant reported for this ion from *n*-pentyl nitrite^53^ and somewhat shorter than the 11 ps reported for *n*-butyl bromide.^17^ The *T*_2_ ∼ 1–1.6 ps for C_3_H_7_^+^ and C_3_H_6_^+^ from *n*-hexane is in rough agreement with the ∼850–880 fs reported for these ions from *n*-butyl bromide. The *T*_1_ ∼ 50–180 fs for C_2_H_5_^+^ and C_2_H_3_^+^ is somewhat faster than the fast time constants of 261 fs for C_2_H_5_^+^ and 557 fs for C_2_H_3_^+^ from *n*-butyl bromide,^17^ whereas the range of *T*_2_ ∼ 1–4 ps generally agrees with the slow time constants for these ions from both *n*-butyl bromide (4–5.5 ps)^17^ and *n*-pentyl nitrite (1.2–2.1 ps).^53^ We note that dynamics in fragment ion yields and competing dissociation pathways on time scales up to 5 ps were also observed in perfluoroalkanes,^52^ but with completely different patterns of competing fragments than observed for the alkanes here.

## 4 Conclusions

This work found that the five C_6_H_14_ isomers undergo distinct dissociation pathways upon SFI that can be rationalized by their calculated cation structures and electronic excited states. The preferential fragmentation pathways upon SFI in each branched isomer are explained by relaxation of the cations to structures containing one elongated C–C bond. Ion yield oscillations observed in 3-methylpentane and 2,3-dimethylbutane are attributed to coherent vibrational excitation along the elongated C–C bond in each isomer. The significantly higher depletion of the *n*-hexane molecular ion compared to the branched isomers upon time-delayed excitation at 713 nm is attributed to a strongly coupled excited state at 2.6 eV in the most populated conformer that can be reached by a two-photon transition. In contrast, the branched isomers only have strongly coupled excited states above 3.9 eV. Competition between dissociative rearrangement and direct C–C bond cleavage reactions was observed in all isomers in the dynamics of fragment ions containing the same number of carbon atoms and different numbers of hydrogen atoms, with extracted time constants on the order of ∼50–300 fs that may correspond to the time needed for hydrogen migration in the metastable cations. Future investigations with molecular dynamics simulations could aid in the assignment of the experimental time constants with particular hydrogen migration or other reactions.

## Supplementary Material



## Notes

Experimental characterization of laser intensity and instrument response function; tabulated computational results of geometries and energy levels; and tabulated coefficients and figures for dynamical fitting of transients ion signals (PDF)
