# Supplementary material for: How Isomer and Conformer Structures Impact Dissociation Dynamics of Alkane Radical Cations
Source: J Phys Chem A. 2025 Sep 8;129(37):8550–62. doi: 10.1021/acs.jpca.5c04290 (PMC12451669; doi:10.1021/acs.jpca.5c04290)
Supplement: Supplementary file 1 [file jp5c04290_si_001.pdf]

Supporting Information: How Isomer and Conformer Structures Impact Dissociation Dynamics of Alkane Radical Cations

Madison Minvielle, Mikaela Aftel, Timothy Hill, Hugo A. López Peña, and Katharine Moore Tibbetts*

Department of Chemistry, Virginia Commonwealth University, Richmond, VA 23284, US

E-mail: kmtibbetts@vcu.edu

Contents

Number of pages: 36

Number of figures: 14

Number of tables: 41

SI Characterization of experimental laser parameters

The intensity of the 1800 nm pump pulse was calibrated by measuring the saturation of ${O_2}^+$ using the experimental method of Hankin[1] and the reported saturation intensity of 2.5×10^{14} W cm^{-2} from the DFT calculations of Usachenko.[2] Under our experimental conditions, the ${O_2}^+$ signal saturated at 105 mW of laser power (Figure S1). Reported intensity of the pump pulse is based on this measurement.

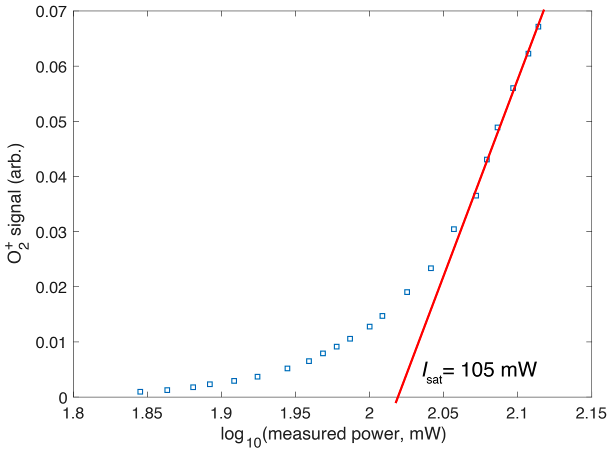

Figure S1: ${O_2}^+$ ion signal measured over a range of pump powers on a semilog scale, with the linear fit used to determine $I_{\text{sat}} = 105$ mW.

To establish the instrument response function (IRF) and the zero-delay temporal overlap between the 1800 nm pump and 713 nm probe pulses, air was leaked into the mass spectrometer and the ${N_2}^+$ signal measured (Figure S2). Fitting this data to a Gaussian function

$$S_{{N_2}^+} = a \exp(-x^2/s^2) \tag{S.1}$$

gave a width parameter $s = 21$ fs used for curve fitting of the transient ion signals, corresponding to a full width at half maximum (FWHM) of 35 fs.

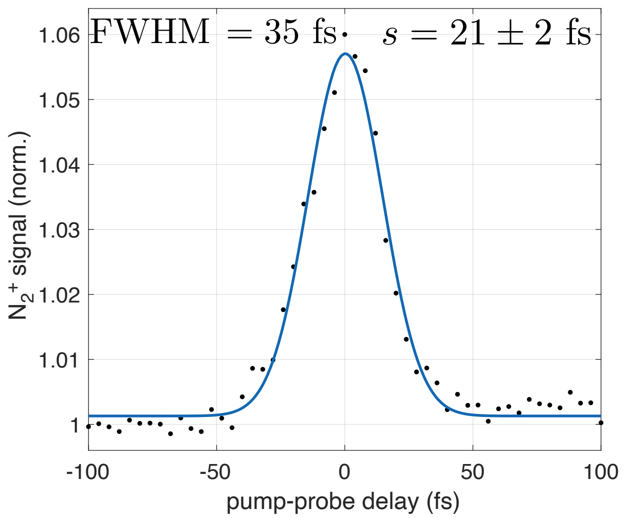

Figure S2: N_2^+ ion signal with fitting to Gaussian function to determine the IRF parameters.

SII Geometries of hexane isomers and conformers

Table S1: XYZ coordinates (in Å) of *n*-hexane conformer 1 optimized geometry.

Atom	X	Y	Z
C	-3.221060	0.220718	-0.000160
C	-1.893690	-0.541280	-0.000190
C	-0.666880	0.376937	0.000044
C	0.666889	-0.376880	-0.000098
C	1.893699	0.541338	0.000157
C	3.221070	-0.220660	-0.000011
H	-4.076270	-0.461420	-0.000320
H	-3.312110	0.862824	-0.882270
H	-3.312230	0.862545	0.882141
H	-1.851860	-1.201330	-0.875750
H	-1.851980	-1.201600	0.875176
H	-0.710580	1.037920	0.876102
H	-0.710540	1.038327	-0.875710
H	0.710600	-1.037860	-0.876160
H	0.710550	-1.038280	0.875650
H	1.851913	1.201303	0.875780
H	1.851948	1.201735	-0.875140
H	3.312121	-0.862940	0.881969
H	4.076276	0.461486	0.000300
H	3.312259	-0.862310	-0.882440

Table S2: XYZ coordinates (in Å) of n-hexane cation conformer 1 optimized geometry.

Atom	X	Y	Z
C	-3.099580	0.258936	-0.000078
C	-1.838310	-0.613060	-0.000280
C	-0.615510	0.454413	0.000027
C	0.615525	-0.454350	-0.000120
C	1.838322	0.613116	0.000145
C	3.099595	-0.258880	0.000031
H	-3.928550	-0.470790	-0.000310
H	-3.196250	0.876172	-0.892640
H	-3.196310	0.875673	0.892825
H	-1.767300	-1.236020	-0.890460
H	-1.767350	-1.236500	0.889562
H	-0.689680	1.072536	0.892830
H	-0.689600	1.072943	-0.892500
H	0.689684	-1.072490	-0.892920
H	0.689624	-1.072870	0.892412
H	1.767299	1.236140	0.890274
H	1.767375	1.236494	-0.889740
H	3.196267	-0.876010	0.892663
H	3.928564	0.470847	0.000172
H	3.196324	-0.875710	-0.892800

Table S3: XYZ coordinates (in Å) of n-hexane conformer 2 optimized geometry.

Atom	X	Y	Z
C	-3.046820	0.149959	-0.319870
C	-1.580780	-0.280380	-0.412460
C	-0.729910	0.238150	0.753415
C	0.729903	-0.238460	0.753366
C	1.580762	0.280289	-0.412410
C	3.046754	-0.150250	-0.320040
H	-3.513550	-0.218400	0.599507
H	-3.631940	-0.231530	-1.161740
H	-3.142710	1.240662	-0.320380
H	-1.164280	0.067378	-1.364310
H	-1.523040	-1.376280	-0.440440
H	-1.200100	-0.072030	1.695429
H	-0.752680	1.336520	0.757614
H	0.752667	-1.336830	0.757358
H	1.200097	0.071539	1.695435
H	1.523149	1.376206	-0.440080
H	1.164155	-0.067140	-1.364330
H	3.631888	0.231516	-1.161770
H	3.142523	-1.240960	-0.321000
H	3.513549	0.217686	0.599473

Table S4: XYZ coordinates (in Å) of n-hexane cation conformer 2 optimized geometry.

Atom	X	Y	Z
C	-3.038626	0.178998	-0.297136
C	-1.650455	-0.286619	-0.523631
C	-0.657245	0.290063	0.881647
C	0.703443	-0.267836	0.697308
C	1.577704	0.23125	-0.445349
C	3.056638	-0.144643	-0.291917
H	-3.539633	-0.299415	0.54596
H	-3.592349	-0.151295	-1.203099
H	-3.147825	1.263146	-0.25208
H	-1.091607	0.202468	-1.321515
H	-1.493595	-1.362923	-0.491263
H	-1.234711	-0.1169	1.710831
H	-0.754713	1.370969	0.788908
H	0.735293	-1.355037	0.81773
H	1.148726	0.117418	1.661935
H	1.470471	1.315594	-0.5534
H	1.203508	-0.220125	-1.377302
H	3.621442	0.17802	-1.167385
H	3.188735	-1.224866	-0.192222
H	3.502432	0.335356	0.582743

Table S5: XYZ coordinates (in Å) of n-hexane conformer 3 optimized geometry.

Atom	X	Y	Z
C	-2.606760	-0.211350	0.859825
C	-2.045350	-0.185000	-0.565280
C	-0.674920	0.495444	-0.700160
C	0.475071	-0.223020	0.015387
C	1.839337	0.437357	-0.211750
C	2.986466	-0.278140	0.505375
H	-1.983210	-0.800420	1.537755
H	-3.609520	-0.648050	0.880915
H	-2.680500	0.799386	1.275418
H	-2.758220	0.333031	-1.217710
H	-1.979000	-1.210590	-0.950650
H	-0.746260	1.526488	-0.327680
H	-0.428820	0.579921	-1.766350
H	0.517597	-1.266480	-0.326280
H	0.277536	-0.267870	1.093204
H	1.795664	1.481996	0.121456
H	2.048708	0.475150	-1.288260
H	3.945359	0.215461	0.322197
H	3.081258	-1.315860	0.169347
H	2.828827	-0.298610	1.588629

Table S6: XYZ coordinates (in Å) of *n*-hexane cation conformer 3 optimized geometry.

Atom	X	Y	Z
C	-2.579080	-0.203370	0.892404
C	-1.995420	-0.264240	-0.487700
C	-0.579070	0.549810	-0.712180
C	0.443708	-0.299920	0.019304
C	1.832560	0.496964	-0.270080
C	2.891484	-0.287340	0.511473
H	-2.097490	-0.880570	1.600818
H	-3.625890	-0.538120	0.827951
H	-2.595740	0.808191	1.303070
H	-2.564580	0.342112	-1.215470
H	-1.890470	-1.275410	-0.878510
H	-0.706470	1.548234	-0.296220
H	-0.399930	0.608382	-1.784250
H	0.547821	-1.305280	-0.386340
H	0.271811	-0.343850	1.092462
H	1.735812	1.522967	0.081872
H	2.022303	0.495554	-1.342330
H	3.832163	0.254393	0.307849
H	3.011895	-1.312530	0.162552
H	2.727828	-0.277150	1.588729

Table S7: XYZ coordinates (in Å) of 2-methylpentane conformer 1 optimized geometry.

Atom	X	Y	Z
C	-2.846095	0.212228	-0.154391
C	-1.499316	-0.362975	0.292053
C	-0.307154	0.479051	-0.177394
C	1.072434	0.018200	0.328375
C	1.450604	-1.379040	-0.181651
C	2.153097	1.039012	-0.051807
H	-2.999474	1.222107	0.239616
H	-3.680699	-0.405612	0.189617
H	-2.910835	0.274159	-1.245589
H	-1.406874	-1.388179	-0.082486
H	-1.480345	-0.438008	1.386995
H	-0.465901	1.517011	0.143801
H	-0.292853	0.507178	-1.276370
H	1.025829	-0.025466	1.426173
H	1.478394	-1.401916	-1.277253
H	0.745162	-2.146056	0.147301
H	2.441572	-1.673848	0.176942
H	1.922922	2.033784	0.341959
H	2.244794	1.128655	-1.140120
H	3.133712	0.747419	0.336752

Table S8: XYZ coordinates (in Å) of 2-methylpentane cation conformer 1 optimized geometry.

Atom	X	Y	Z
C	-2.892570	0.249676	-0.165354
C	-1.524254	-0.384091	0.286853
C	-0.483370	0.533735	-0.218981
C	1.285964	-0.051991	0.380886
C	1.486002	-1.415231	-0.180555
C	2.169813	1.055904	-0.078785
H	-3.051889	1.232679	0.277566
H	-3.676018	-0.424716	0.191057
H	-2.965955	0.325311	-1.250215
H	-1.447360	-1.383900	-0.140023
H	-1.527292	-0.453811	1.376829
H	-0.448281	1.526310	0.223995
H	-0.277566	0.526175	-1.287857
H	1.014344	-0.018465	1.437181
H	1.550100	-1.427835	-1.271100
H	0.755907	-2.144570	0.166351
H	2.467751	-1.760568	0.187746
H	1.875339	2.037424	0.293442
H	2.303493	1.084090	-1.161797
H	3.164815	0.851578	0.355284

Table S9: XYZ coordinates (in Å) of 2-methylpentane conformer 2 optimized geometry.

Atom	X	Y	Z
C	-2.292893	0.282834	0.759882
C	-1.541555	0.530274	-0.551913
C	-0.325592	-0.383000	-0.773112
C	0.818159	-0.274554	0.254135
C	1.902083	-1.317797	-0.048501
C	1.425780	1.133311	0.307960
H	-1.680706	0.513343	1.636300
H	-2.607451	-0.762729	0.846395
H	-3.192553	0.902014	0.821942
H	-1.237370	1.581644	-0.607304
H	-2.233210	0.382354	-1.389729
H	0.086835	-0.183881	-1.771754
H	-0.672631	-1.424716	-0.797176
H	0.411144	-0.504199	1.247720
H	2.357155	-1.140695	-1.029583
H	1.492371	-2.332599	-0.054583
H	2.704264	-1.288881	0.695325
H	0.695945	1.889822	0.607844
H	1.822945	1.427508	-0.670437
H	2.252238	1.177983	1.023818

Table S10: XYZ coordinates (in Å) of 2-methylpentane cation conformer 2 optimized geometry.

Atom	X	Y	Z
C	-2.318491	0.282694	0.778227
C	-1.579072	0.534522	-0.533721
C	-0.486924	-0.388363	-0.901131
C	1.003836	-0.253433	0.362616
C	1.927663	-1.337008	-0.070202
C	1.471308	1.157234	0.312642
H	-1.709401	0.573256	1.640252
H	-2.600654	-0.766413	0.893773
H	-3.230162	0.879485	0.822082
H	-1.299191	1.582165	-0.668652
H	-2.285592	0.339982	-1.377700
H	0.089585	-0.155043	-1.794413
H	-0.673480	-1.450706	-0.758882
H	0.356258	-0.504081	1.205893
H	2.443880	-1.117504	-1.007020
H	1.460001	-2.320793	-0.116050
H	2.709442	-1.405670	0.706852
H	0.706302	1.884442	0.580814
H	1.933040	1.424542	-0.640811
H	2.266613	1.248730	1.072660

Table S11: XYZ coordinates (in Å) of 3-methylpentane optimized geometry.

Atom	X	Y	Z
C	-2.584556	0.171313	0.286785
C	-1.263173	0.664813	-0.310056
C	-0.000022	-0.052467	0.205110
C	0.000578	-1.543506	-0.159358
C	1.262812	0.665710	-0.309572
C	2.584430	0.172351	0.286865
H	-2.571805	0.225346	1.380590
H	-2.805584	-0.862875	0.010648
H	-3.422351	0.782910	-0.060586
H	-1.162634	1.736630	-0.099872
H	-1.301368	0.577933	-1.404341
H	-0.000246	0.026940	1.303105
H	-0.877018	-2.060067	0.236500
H	0.877884	-2.059648	0.237691
H	0.001344	-1.679368	-1.247339
H	1.161788	1.737331	-0.098628
H	1.301018	0.579616	-1.403921
H	2.571537	0.225135	1.380732
H	3.421884	0.784856	-0.059725
H	2.806103	-0.861389	0.009573

Table S12: XYZ coordinates (in Å) of 3-methylpentane cation optimized geometry.

Atom	X	Y	Z
C	-2.635657	0.176316	0.286311
C	-1.440013	0.776312	-0.338761
C	0.184533	-0.176700	0.253314
C	-0.000493	-1.589095	-0.173151
C	1.278730	0.641936	-0.342353
C	2.636128	0.193967	0.299494
H	-2.630430	0.211361	1.377746
H	-2.876948	-0.823704	-0.070471
H	-3.473394	0.831778	-0.024473
H	-1.143706	1.760240	0.013508
H	-1.288857	0.621392	-1.404609
H	-0.011436	0.016621	1.310364
H	-0.886484	-2.061502	0.248729
H	0.858088	-2.153995	0.228720
H	0.023681	-1.719227	-1.257385
H	1.151715	1.704415	-0.123649
H	1.335031	0.511770	-1.425520
H	2.622772	0.281366	1.387002
H	3.411431	0.859229	-0.085695
H	2.885927	-0.830914	0.025081

Table S13: XYZ coordinates (in Å) of 2,3-dimethylbutane optimized geometry.

Atom	X	Y	Z
C	-1.008725	1.195224	-0.853886
C	-0.712180	0.310566	0.365925
C	-1.806107	-0.753790	0.533009
C	0.712168	-0.310531	0.365990
C	1.008787	-1.195270	-0.853743
C	1.806082	0.753836	0.533077
H	-0.267826	1.988466	-0.982825
H	-1.984519	1.678996	-0.748463
H	-1.037739	0.612560	-1.779730
H	-0.757225	0.956272	1.254292
H	-2.787114	-0.287960	0.667878
H	-1.620254	-1.388049	1.405620
H	-1.878696	-1.406824	-0.342091
H	0.757155	-0.956177	1.254404
H	1.037920	-0.612658	-1.779617
H	0.267859	-1.988480	-0.982711
H	1.984546	-1.679086	-0.748199
H	2.787091	0.288018	0.667974
H	1.620203	1.388108	1.405673
H	1.878683	1.406856	-0.342033

Table S14: XYZ coordinates (in Å) of 2,3-dimethylbutane cation optimized geometry.

Atom	X	Y	Z
C	-1.073846	1.232882	-0.865637
C	-0.984691	0.401942	0.360115
C	-1.872947	-0.776490	0.522434
C	0.984676	-0.401911	0.360201
C	1.073909	-1.232922	-0.865497
C	1.872922	0.776531	0.522507
H	-0.289685	1.985311	-0.945399
H	-2.022147	1.792816	-0.787005
H	-1.139666	0.646330	-1.783174
H	-0.746836	0.935898	1.278879
H	-2.871169	-0.376629	0.770799
H	-1.597909	-1.421552	1.358315
H	-1.991622	-1.362466	-0.389605
H	0.746763	-0.935814	1.278981
H	1.139786	-0.646423	-1.783064
H	0.289753	-1.985355	-0.945265
H	2.022205	-1.792851	-0.786774
H	2.871128	0.376684	0.770958
H	1.597831	1.421641	1.358334
H	1.991654	1.362454	-0.389558

Table S15: XYZ coordinates (in Å) of 2,2-dimethylbutane optimized geometry.

Atom	X	Y	Z
C	-2.195308	0.012692	-0.000088
C	-0.886666	0.809808	-0.000147
C	0.433589	-0.003278	0.000026
C	0.533847	-0.885901	1.257165
C	0.533814	-0.886480	-1.256709
C	1.605740	0.995076	-0.000217
H	-2.290891	-0.625098	-0.882940
H	-3.053001	0.691725	-0.000144
H	-2.290903	-0.624964	0.882859
H	-0.878468	1.473033	0.874234
H	-0.878400	1.472764	-0.874732
H	1.498285	-1.402500	1.294467
H	-0.245094	-1.652586	1.283798
H	0.444887	-0.288495	2.170740
H	1.498273	-1.403052	-1.293830
H	0.444767	-0.289505	-2.170557
H	-0.245095	-1.653213	-1.282933
H	1.578096	1.641426	0.883129
H	2.569438	0.475733	-0.000094
H	1.578092	1.641000	-0.883874

Table S16: XYZ coordinates (in Å) of 2,2-dimethylbutane cation optimized geometry.

Atom	X	Y	Z
C	-2.290734	0.030754	0.000031
C	-1.120085	0.924819	0.000177
C	0.707938	-0.150012	-0.000031
C	0.577614	-0.916837	1.276622
C	0.577569	-0.916418	-1.276932
C	1.646757	1.015246	0.000143
H	-2.384796	-0.578853	-0.899007
H	-3.168499	0.705360	0.000166
H	-2.384772	-0.579179	0.898850
H	-0.885431	1.460829	0.914783
H	-0.885451	1.461153	-0.914243
H	1.519818	-1.474498	1.403187
H	-0.219053	-1.659817	1.256856
H	0.480926	-0.279717	2.156907
H	1.519771	-1.474034	-1.403714
H	0.480846	-0.279010	-2.157004
H	-0.219095	-1.659408	-1.257379
H	1.567561	1.634384	0.894454
H	2.666563	0.598709	0.000061
H	1.567535	1.634672	-0.893966

SIII QTAIM analysis of cationic hexane isomers

Critical points in the context of QTAIM anlysis are classified according to their rank (ω) and signature (σ) and are symbolized by (ω, σ). The rank is the number of non-zero curvatues of ρ at the critical point. The signature is the algebraic sum of the signs of the curvatures, i.e., each of the three curvatures contributes ± 1 depending on wether it is positive or negative curvature. There are four types of stable critical points having three non-zero eigenvalues: (3,-3) or nuclear critical point (NCP); (3,-1) or bond critical point (BCP); (3,+1) or ring critical point (RCP); and (3,+3) or cage critical point (CCP). For the system of interest in this work, we only have NCPs and BCPs.

It is important to note that the numbering of the carbon atoms in Figure 2 of the main text was done for convenience and to make the discussion clear and concise however, it does not necessarily match the numeration in the original inputs/outputs of the computational calculations. In order to clarify this, Tables S17 to S20 contain lines in bold letters emphasizing the BCPs relevant in the discussion of the elongated C2–C3 bonds mentioned in the main text. For example, in the case of 2-methylpentane cation, it can be found on Table S17 that the critical point with index 9, actually corresponds to the BCP joining carbon atoms C3 and C4 (shown in the "Description" column as "3(C)>4(C)").

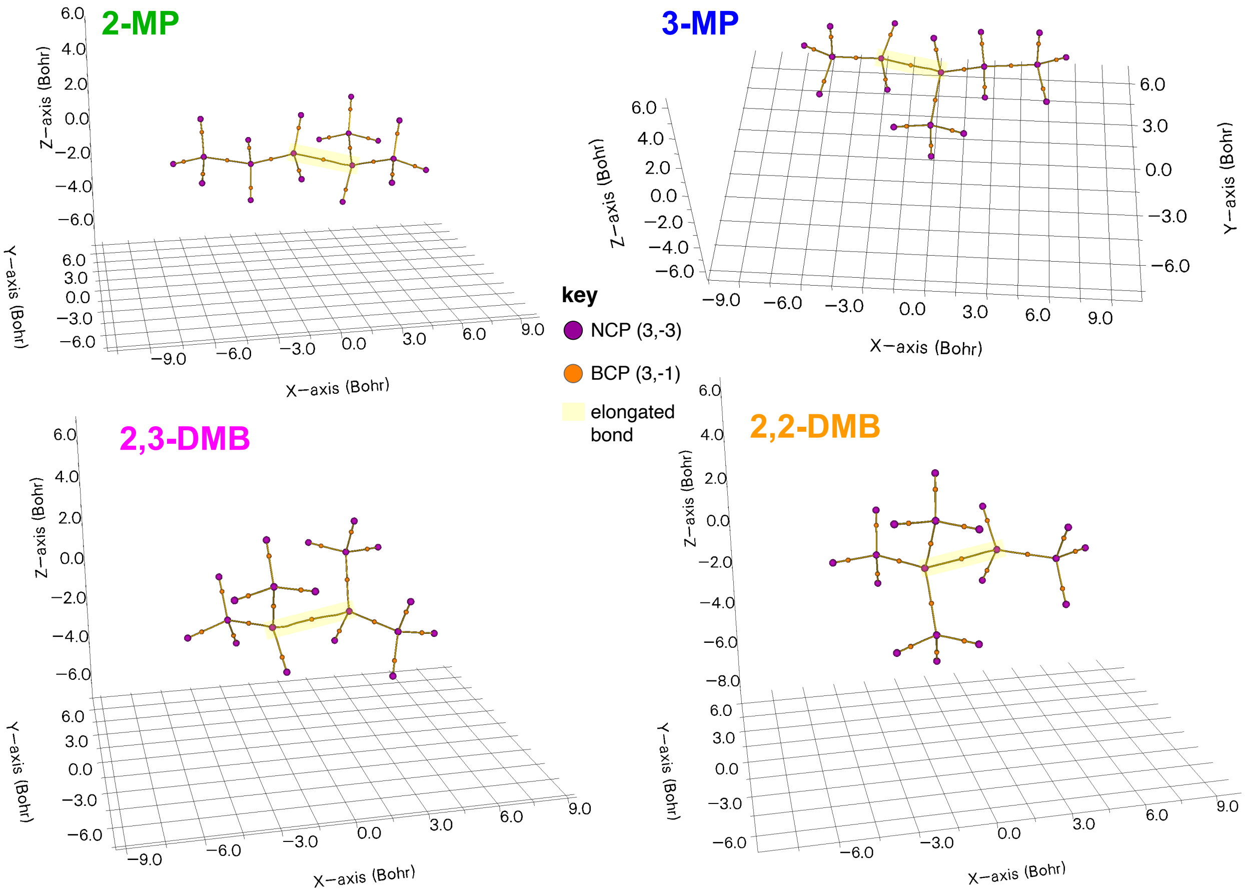

Figure S3: Molecular graphs including nuclear critical points (NCPs), bond critical points (BCPs), and bond paths connecting nuclei sharing a BCP for the cationic hexane isomers 2-MP$^+$, 3-MP$^+$, 2,3-DMB$^+$, and 2,2-DMB$^+$.

Table S17: XYZ coordinates (in Bohr) for the critical points found for 2-methylpentane cation conformer 1. The critical point relevant for the discussion is shown in bold letters.

Index	X	Y	Z	Type	Description
1	3.68451605	-3.24760153	-0.40034935	BCP	18(H)> 6(C)
2	-0.89750515	-2.22854277	-0.22517377	BCP	12(H)> 3(C)
3	-5.67582464	-1.58444182	-0.31982422	BCP	7(H)>1(C)
4	4.2217026	-2.13221147	1.42372785	BCP	19(H)>6(C)
5	-5.56191578	-0.5241539	1.58518953	BCP	9(H)>1(C)
6	-0.67412609	-1.03493267	1.69732955	BCP	13(H)> 3(C)
7	5.29284098	-1.81044676	-0.42988484	BCP	6(C)>20(H)
8	3.28260149	-1.0599763	-0.28000985	BCP	6(C)>4(C)
**9**	**0.75142192**	**-0.45184779**	**-0.18870091**	**BCP**	**3(C)>4(C)**
10	-4.20026641	0.16289836	-0.12968325	BCP	1(C)>2(C)
11	-6.41482483	0.46195068	-0.16364752	BCP	1(C)>8(H)
12	-1.93714958	-0.04781696	-0.12830288	BCP	3(C)>2(C)
13	2.095787	0.07417958	-2.04205841	BCP	4(C)>14(H)
14	-2.85996087	0.89772287	-1.88618075	BCP	2(C)>11(H)
15	-2.75215863	1.9891361	0.01004189	BCP	2(C)>10(H)
16	2.63811806	1.41505698	-0.11443406	BCP	4(C)>5(C)
17	2.91971402	2.58667183	1.74606957	BCP	15(H)>5(C)
18	4.06251257	3.02502795	-0.03087549	BCP	5(C)>17(H)
19	1.96417447	3.53090995	0.00008184	BCP	5(C)>16(H)
20	3.49390511	-3.86928325	-0.65278614	NCP	Nucleus: 18(H)
21	-0.88931244	-2.83797265	-0.5116225	NCP	Nucleus: 12(H)
22	-5.79509179	-2.20583514	-0.61902774	NCP	Nucleus: 7(H)
23	4.30797571	-2.17672214	2.11503851	NCP	Nucleus: 19(H)
24	-5.621847	-0.5927695	2.27917515	NCP	Nucleus: 9(H)
25	-0.55521641	-1.05467153	2.36033989	NCP	Nucleus: 13(H)
26	4.06891245	-2.06549403	0.09430807	NCP	Nucleus: 6(C)
27	-0.93025797	-1.00860708	0.36488489	NCP	Nucleus: 3(C)
28	-5.47365879	-0.39864447	0.25899202	NCP	Nucleus: 1(C)
29	5.93660781	-1.69045935	-0.69798886	NCP	Nucleus: 20(H)
30	-6.91355484	0.90083798	-0.37809451	NCP	Nucleus: 8(H)
31	2.43517728	0.07667657	-0.72391547	NCP	Nucleus: 4(C)
32	-2.86583332	0.78154201	-0.55160091	NCP	Nucleus: 2(C)
33	1.93644989	0.07563099	-2.69782002	NCP	Nucleus: 14(H)
34	-2.85975542	0.96692523	-2.58221414	NCP	Nucleus: 11(H)
35	-2.69697569	2.62302995	0.29667696	NCP	Nucleus: 10(H)
36	2.95836039	2.58263018	2.4449682	NCP	Nucleus: 15(H)
37	2.84789495	2.61680078	0.40857516	NCP	Nucleus: 5(C)
38	4.69448114	3.25138464	-0.25386941	NCP	Nucleus: 17(H)
39	1.50893449	4.016136	-0.20749852	NCP	Nucleus: 16(H)

Table S18: XYZ coordinates (in Bohr) for the critical points found for 3-methylpentane cation conformer 1. The critical point relevant for the discussion is shown in bold letters.

Index	X	Y	Z	Type	Description
1	-1.08978148	-3.57547875	0.19357475	BCP	13(H)>4(C)
2	1.05635992	-3.68758045	0.16893647	BCP	14(H)>4(C)
3	0.03020261	-3.15492713	-1.65785579	BCP	4(C)>15(H)
4	0.1510317	-1.7368052	0.04261064	BCP	4(C)>3(C)
5	-5.27105933	-0.89541414	0.09880665	BCP	8(H)>1(C)
6	-4.96804246	0.37999974	1.88168164	BCP	7(H)>1(C)
7	0.09908494	-0.08995268	1.79423042	BCP	12(H)>3(C)
8	5.27672753	-0.88653813	0.22806583	BCP	20(H)>6(C)
9	-3.90026065	0.85082852	-0.00738494	BCP	1(C)>2(C)
10	-6.00406586	1.14433381	0.15505304	BCP	1(C)>9(H)
**11**	**-1.18250332**	**0.56526002**	**-0.07988423**	**BCP**	**3(C)>2(C)**
12	4.95605541	0.47456854	1.89535211	BCP	18(H)>6(C)
13	1.43409728	0.49052053	-0.12905511	BCP	3(C)>5(C)
14	3.7282549	0.76333295	-0.01418283	BCP	6(C)>5(C)
15	-2.52794078	1.2676797	-1.96946437	BCP	2(C)>11(H)
16	5.92793263	1.18819932	0.08856966	BCP	6(C)>19(H)
17	2.48217723	1.04765185	-1.973999	BCP	5(C)>17(H)
18	-2.34630036	2.69188116	-0.19584432	BCP	2(C)>10(H)
19	2.25660742	2.5119318	-0.37366792	BCP	5(C)>16(H)
20	-1.65499764	-3.88447995	0.46046281	NCP	Nucleus: 13(H)
21	1.60283488	-4.05767989	0.42349575	NCP	Nucleus: 14(H)
22	-0.00093107	-3.00295317	-0.32720741	NCP	Nucleus: 4(C)
23	0.0442319	-3.24552954	-2.35146234	NCP	Nucleus: 15(H)
24	-5.43076241	-1.53350504	-0.12508399	NCP	Nucleus: 8(H)
25	5.4475563	-1.54712047	0.05354978	NCP	Nucleus: 20(H)
26	-4.97056899	0.39874106	2.57857613	NCP	Nucleus: 7(H)
27	-0.01712241	0.02691209	2.45107018	NCP	Nucleus: 12(H)
28	0.3487138	-0.33391351	0.47869366	NCP	Nucleus: 3(C)
29	-4.98066887	0.33319002	0.54104865	NCP	Nucleus: 1(C)
30	4.95621557	0.52972781	2.59644999	NCP	Nucleus: 18(H)
31	-6.54525108	1.55774934	-0.03963569	NCP	Nucleus: 9(H)
32	4.98155741	0.366545	0.56596086	NCP	Nucleus: 6(C)
33	-2.72122734	1.4670155	-0.64016464	NCP	Nucleus: 2(C)
34	-2.4390661	1.17784352	-2.62854104	NCP	Nucleus: 11(H)
35	2.41644879	1.21308226	-0.64695246	NCP	Nucleus: 5(C)
36	2.52124083	0.9698354	-2.66918866	NCP	Nucleus: 17(H)
37	6.42883687	1.60868107	-0.15328962	NCP	Nucleus: 19(H)
38	-2.16828352	3.30244584	0.01708928	NCP	Nucleus: 10(H)
39	2.17890701	3.1967183	-0.23844245	NCP	Nucleus: 16(H)

Table S19: XYZ coordinates (in Bohr) for the critical points found for 2,2-dimethylbutane cation. The critical point relevant for the discussion is shown in bold letters.

Index	X	Y	Z	Type	Description
1	-0.23272637	-2.61024507	2.3810031	BCP	17(H)>5(C)
2	-2.35639477	-2.27707606	2.55903367	BCP	15(H)>5(C)
3	-0.23268078	-2.6103228	-2.38090522	BCP	13(H)> 4(C)
4	-1.00830989	-0.87438278	3.48611671	BCP	5(C)>16(H)
5	-2.35634206	-2.27714501	-2.55899959	BCP	12(H)> 4(C)
6	-1.23077686	-0.96172205	1.27791925	BCP	5(C)>3(C)
7	-1.2307488	-0.96176026	-1.27789689	BCP	4(C)>3(C)
8	4.40997534	-0.88436137	1.10568686	BCP	7(H)>1(C)
9	4.40998767	-0.88433447	-1.10570418	BCP	9(H)>1(C)
10	-1.00822756	-0.8744877	-3.48608988	BCP	4(C)>14(H)
11	-2.20479135	1.02385121	-0.00002992	BCP	3(C)>6(C)
**12**	**0.41827562**	**0.71444943**	**0.00000366**	**BCP**	**3(C)>2(C)**
13	3.31900872	0.69664962	0.00000444	BCP	1(C)>2(C)
14	-4.27384141	1.63171133	-0.00005095	BCP	19(H)>6(C)
15	5.45003909	0.64753361	0.00001849	BCP	1(C)>8(H)
16	-2.86253676	2.83792005	1.09867807	BCP	6(C)>20(H)
17	1.94662316	2.33916873	1.14211186	BCP	2(C)>11(H)
18	-2.86252453	2.83787698	-1.09881421	BCP	6(C)>18(H)
19	1.94662555	2.33918138	-1.14208821	BCP	2(C)>10(H)
20	0.25221456	-3.11388611	2.37576509	NCP	Nucleus: 17(H)
21	-2.97407934	-2.60877743	2.64898956	NCP	Nucleus: 15(H)
22	-1.16510165	-1.65541275	2.41277857	NCP	Nucleus: 5(C)
23	0.25225628	-3.11396725	-2.37563947	NCP	Nucleus: 13(H)
24	-2.97402645	-2.60884549	-2.64896103	NCP	Nucleus: 12(H)
25	-0.92612448	-0.47563581	4.05576331	NCP	Nucleus: 16(H)
26	4.45610376	-1.27990213	1.67820213	NCP	Nucleus: 7(H)
27	-1.16504874	-1.65548456	-2.41273322	NCP	Nucleus: 4(C)
28	-1.3397584	-0.19639423	-0.00000189	NCP	Nucleus: 3(C)
29	4.45612243	-1.27986104	-1.67822823	NCP	Nucleus: 9(H)
30	4.33712847	-0.13439411	0	NCP	Nucleus: 1(C)
31	-0.92602677	-0.47575805	-4.05574593	NCP	Nucleus: 14(H)
32	-4.94363213	1.4068291	-0.00004919	NCP	Nucleus: 19(H)
33	-3.0031807	2.09036846	-0.0000548	NCP	Nucleus: 6(C)
34	6.03654034	1.04367375	0.00002802	NCP	Nucleus: 8(H)
35	2.21079203	1.66201117	0.00000945	NCP	Nucleus: 2(C)
36	1.82260358	2.68244948	1.70612424	NCP	Nucleus: 11(H)
37	-2.79815169	3.23761866	1.66947815	NCP	Nucleus: 20(H)
38	-2.79813303	3.23755329	-1.66962878	NCP	Nucleus: 18(H)
39	1.82260729	2.68246819	-1.70609797	NCP	Nucleus: 10(H)

Table S20: XYZ coordinates (in Bohr) for the critical points found for 2,3-dimethylbutane cation conformer 1. The critical point relevant for the discussion is shown in bold letters.

Index	X	Y	Z	Type	Description
1	-1.19673685	-3.2031741	1.69741351	BCP	7(H)>1(C)
2	-3.31530502	-2.87948	1.50107852	BCP	8(H)>1(C)
3	-2.17339055	-1.51686469	2.72683428	BCP	1(C)> 9(H)
4	3.58778259	-2.32929779	0.10437684	BCP	20(H)>6(C)
5	-1.99771195	-1.49925779	0.520418	BCP	1(C)>2(C)
6	3.10114732	-2.38177337	-2.04308552	BCP	19(H)> 6(C)
7	-1.6152639	-1.35790247	-1.85527256	BCP	10(H)>2(C)
8	4.71766419	-1.16118093	-1.32154674	BCP	6(C)>18(H)
9	2.71239164	-0.53952063	-0.86495705	BCP	6(C)> 4(C)
**10**	**-0.0000238**	**-0.00001914**	**-0.71734911**	**BCP**	**2(C)>4(C)**
11	-2.71244966	0.53947238	-0.86480917	BCP	2(C)>3(C)
12	-4.71775042	1.16110765	-1.32130289	BCP	11(H)>3(C)
13	2.17356847	1.51701575	2.72660681	BCP	15(H)>5(C)
14	1.99774618	1.49928745	0.52020314	BCP	4(C)>5(C)
15	-3.58777693	2.32930315	0.10448301	BCP	3(C)>13(H)
16	1.61514191	1.35779992	-1.85545446	BCP	4(C)>14(H)
17	-3.10128164	2.38165944	-2.04301395	BCP	3(C)>12(H)
18	3.31540414	2.87956225	1.50070238	BCP	5(C)>17(H)
19	1.19684853	3.20326749	1.69715665	BCP	5(C)>16(H)
20	-0.71711931	-3.70813303	1.75462977	NCP	Nucleus: 7(H)
21	-3.93513611	-3.21748531	1.45880133	NCP	Nucleus: 8(H)
22	-2.12268422	-2.24503185	1.60600138	NCP	Nucleus: 1(C)
23	3.65302565	-2.71244647	0.68568218	NCP	Nucleus: 20(H)
24	-2.20069308	-1.14559048	3.3187672	NCP	Nucleus: 9(H)
25	2.91379612	-2.79286981	-2.57769585	NCP	Nucleus: 19(H)
26	-1.48731111	-1.6964109	-2.42449268	NCP	Nucleus: 10(H)
27	3.47645687	-1.61065766	-1.01720939	NCP	Nucleus: 6(C)
28	-1.89025262	-0.6829669	-0.71033296	NCP	Nucleus: 2(C)
29	5.3695918	-0.94021387	-1.48120467	NCP	Nucleus: 18(H)
30	2.20090904	1.1457745	3.31855866	NCP	Nucleus: 15(H)
31	-5.36968773	0.94013291	-1.48090607	NCP	Nucleus: 11(H)
32	1.89020537	0.68292911	-0.71049548	NCP	Nucleus: 4(C)
33	-3.4765249	1.61059908	-1.01707144	NCP	Nucleus: 3(C)
34	2.12279005	2.24512067	1.60573682	NCP	Nucleus: 5(C)
35	-3.65298167	2.71248501	0.68577165	NCP	Nucleus: 13(H)
36	1.48715193	1.69627608	-2.42468499	NCP	Nucleus: 14(H)
37	-2.91396497	2.79272722	-2.57765876	NCP	Nucleus: 12(H)
38	3.93523256	3.21756477	1.45836681	NCP	Nucleus: 17(H)
39	0.71723446	3.70822931	1.75437643	NCP	Nucleus: 16(H)

SIV Population analysis of conformers

Table S21: Relative energy and population analysis (%) of *n*-hexane neutral conformers.

Conformer	Rel. energy (eV)	Population
1	0.0000	71.8
2	0.0427	13.6
3	0.0409	14.6

Table S22: Relative energy and population analysis (%) of 2-methylpentane neutral conformers.

Conformer	Rel. energy (eV)	Population
1	0.0000	82.9
2	0.0405	17.1

SV Ionization Energies

Vertical and adiabatic ionization energies (IE_{vert} and IE_{ad}, respectively) were determined at the B3LYP/6-311+G(d) level of theory.

Table S23: Ionization Energies for n-hexane conformers.

Conformer	IE_{vert} (eV)	IE_{ad} (eV)
1	10.49	9.63
2	10.55	9.78
3	10.49	9.65
Weighted Average	10.5	

Table S24: Ionization Energies of 2-methylpentane conformers.

Conformer	IE_{vert} (eV)	IE_{ad} (eV)
1	10.52	9.54
2	10.49	9.49
Weighted Average	10.52	

Table S25: Ionization Energies of 3-methylpentane, 2,3-dimethylbutane, and 2,2-dimethylbutane.

Molecule	IE_{vert} (eV)	IE_{ad} (eV)
3-MP	10.84	9.52
23-DMB	10.49	9.21
22-DMB	10.56	9.30

SVI TDDFT energy levels

Table S26: Energy levels and oscillator strengths for *n*-hexane cation conformer 1 at S_0 and D_0 geometries. All energies relative to the ground state energy of the D_0 structure.

Conformer 1	Vertical		Adiabatic	
State	Energy (eV)	Oscillator Strength	Energy (eV)	Oscillator Strength
D0	1.1652		0	
D1	1.2768	0	2.5153	0
D2	1.4356	0.0182	2.5639	0
D3	1.6824	0	2.6034	0.2845
D4	2.2145	0	2.7739	0
D5	2.7351	0	3.025	0
D6	2.7552	0	3.2362	0.2549
D7	3.4844	0.0015	3.8117	0
D8	3.9798	0.0001	4.3918	0

Table S27: Energy levels and oscillator strengths for *n*-hexane cation conformer 2 at S_0 and D_0 geometries. All energies relative to the ground state energy of the D_0 structure.

Conformer 2	Vertical		Adiabatic	
State	Energy (eV)	Oscillator Strength	Energy (eV)	Oscillator Strength
D0	0.7746		0.0000	
D1	0.8571	0.0006	2.3055	0.0052
D2	1.0104	0.0000	2.4300	0.0250
D3	1.3556	0.0002	2.6316	0.0310
D4	1.9759	0.1143	3.0667	0.0150
D5	2.0904	0.0016	3.4770	0.0072
D6	2.2809	0.0306	3.6514	0.0268
D7	3.1468	0.0077	4.1929	0.0997
D8	3.5896	0.0033	4.7383	0.0135

Table S28: Energy levels and oscillator strengths for *n*-hexane cation conformer 3 at S_0 and D_0 geometries. All energies relative to the ground state energy of the D_0 structure.

Conformer 3	Vertical		Adiabatic	
State	Energy (eV)	Oscillator Strength	Energy (eV)	Oscillator Strength
D0	0.7813		0.0000	
D1	1.0642	0.0001	2.4112	0.0096
D2	1.1839	0.0003	2.5043	0.1789
D3	1.4516	0.0005	2.6296	0.0488
D4	1.8214	0.0018	2.6705	0.0359
D5	2.3204	0.0607	3.0631	0.0635
D6	2.6371	0.1164	3.4705	0.0187
D7	3.0858	0.0094	4.0225	0.0041
D8	3.6987	0.0004	4.4238	0.0752

Table S29: Energy levels and oscillator strengths for 2-methylpentane cation conformer 1 at S_0 and D_0 geometries. All energies relative to the ground state energy of the D_0 structure.

Conformer 1	Vertical		Adiabatic	
State	Energy (eV)	Oscillator Strength	Energy (eV)	Oscillator Strength
D0	1.3139		0	
D1	1.8071	0.0244	3.2626	0.0012
D2	2.0785	0.0065	3.3569	0.0135
D3	2.5411	0.0022	3.5871	0.0011
D4	3.0374	0.0034	3.8998	0.0038
D5	3.1821	0.0109	4.0148	0.0974
D6	3.5437	0.007	4.5263	0.0377
D7	4.0919	0.0035	4.8114	0.0497
D8	4.7155	0.003	5.2205	0.0575

Table S30: Energy levels and oscillator strengths for 2-methylpentane cation conformer 2 at S_0 and D_0 geometries. All energies relative to the ground state energy of the D_0 structure.

Conformer 2	Vertical		Adiabatic	
State	Energy (eV)	Oscillator Strength	Energy (eV)	Oscillator Strength
D0	0.9771		0	
D1	1.1656	0	3.3743	0.0007
D2	1.3032	0.0001	3.4309	0.0008
D3	1.9744	0.0003	3.5834	0.0169
D4	2.1583	0.0002	3.8833	0.0171
D5	2.5573	0.0632	3.914	0.0385
D6	3.1259	0.0814	4.6413	0.0513
D7	3.2619	0.0097	5.0823	0.0059
D8	3.8173	0.0084	5.4098	0.0191

Table S31: Energy levels and oscillator strengths for 3-methylpentane cation at S_0 and D_0 geometries. All energies relative to the ground state energy of the D_0 structure.

Conformer 1	Vertical		Adiabatic	
State	Energy (eV)	Oscillator Strength	Energy (eV)	Oscillator Strength
D0	1.3068		0	
D1	1.8489	0.0178	3.7044	0.0019
D2	2.2026	0.0151	3.939	0.0495
D3	2.6903	0.0104	4.3781	0.0047
D4	2.8574	0.0008	4.6621	0.0333
D5	3.2923	0.0002	4.7924	0.0625
D6	3.4991	0.0003	5.2261	0.0818
D7	4.1610	0.0121	5.7042	0.0078
D8	4.6142	0	6.1587	0.0027

Table S32: Energy levels and oscillator strengths for 2,3-dimethylbutane cation at S_0 and D_0 geometries. All energies relative to the ground state energy of the D_0 structure.

Conformer 1	Vertical		Adiabatic	
State	Energy (eV)	Oscillator Strength	Energy (eV)	Oscillator Strength
D0	1.1882		0	
D1	1.3943	0	4.1253	0.0005
D2	1.4298	0.0001	4.1312	0.0001
D3	2.3598	0.0010	4.4746	0.0002
D4	2.4064	0	4.5027	0.0001
D5	3.3158	0.0186	5.2481	0.0249
D6	3.3659	0.0115	5.3372	0.3807
D7	3.7128	0.0760	5.3957	0.0609
D8	3.8943	0.0006	5.7669	0.0668

Table S33: Energy levels and oscillator strengths for 2,2-dimethylbutane cation at S_0 and D_0 geometries. All energies relative to the ground state energy of the D_0 structure.

Conformer 1	Vertical		Adiabatic	
State	Energy (eV)	Oscillator Strength	Energy (eV)	Oscillator Strength
D0	1.1620		0.0000	
D1	1.2787	0.0000	4.0425	0.0006
D2	1.3545	0.0000	4.0706	0.0004
D3	2.2831	0.0003	4.2887	0.0003
D4	2.6721	0.0038	4.8879	0.0056
D5	2.7510	0.0001	5.0048	0.0002
D6	3.2894	0.0152	5.3043	0.0344
D7	3.5065	0.0427	5.3944	0.0598
D8	3.9157	0.0594	5.4170	0.3317

SVII Frequency analysis

Table S34: Vibrational frequencies for 3-methylpentane at the relaxed cation (D_0) geometry.

Mode	Frequency	IR Intensity	Mode	Frequency	IR Intensity
1	56.6082	0.3873	28	1295.5081	46.7941
2	77.3288	0.1215	29	1368.7575	48.7176
3	173.6287	2.9835	30	1375.9913	8.572
4	191.9653	0.8442	31	1397.4179	32.9387
5	200.0586	4.054	32	1412.7374	9.3285
6	219.8103	0.0843	33	1430.5316	29.2139
7	238.4072	0.6985	34	1459.6792	12.1012
8	280.7346	0.6808	35	1481.0789	12.2121
9	326.6127	1.1175	36	1483.8775	16.6266
10	419.2601	0.2254	37	1492.3372	0.5242
11	470.689	2.7844	38	1506.795	9.2414
12	702.0255	1.7649	39	1510.3289	4.9406
13	796.3745	4.3528	40	1521.3205	14.7657
14	839.9667	14.6556	41	2926.7781	99.9445
15	850.8674	4.7514	42	2968.9338	29.3061
16	863.0621	25.0249	43	3050.8986	3.345
17	876.6506	2.7867	44	3058.8106	2.6593
18	893.8786	169.4859	45	3080.3719	0.826
19	970.1817	31.7089	46	3082.4438	1.0833
20	996.801	11.8822	47	3096.799	0.7537
21	1061.7857	47.1794	48	3113.0754	6.436
22	1116.6035	31.7586	49	3117.37	16.815
23	1118.536	6.6114	50	3126.8507	9.3553
24	1184.9966	58.2639	51	3143.7223	10.5011
25	1206.3713	2.3846	52	3147.2541	3.0589
26	1237.756	0.5237	53	3154.0344	3.9697
27	1278.4311	3.6256	54	3214.7279	0.2572

Table S35: Vibrational frequencies for 2,3-dimethylbutane at the relaxed cation (D_0) geometry.

Mode	Frequency	IR Intensity	Mode	Frequency	IR Intensity
1	45.9128	0.0691	28	1375.2921	7.5954
2	179.4933	0.896	29	1391.6322	48.3894
3	185.4186	0.051	30	1393.5313	24.4291
4	200.6491	0.15	31	1398.9089	80.5546
5	212.7171	0.7817	32	1413.9564	21.7947
6	224.26	0.0818	33	1450.202	2.5897
7	227.8413	0.6424	34	1463.0775	5.1278
8	233.378	0.0019	35	1463.9056	33.5392
9	350.1361	0.1192	36	1473.2578	15.7803
10	361.1706	1.4679	37	1490.9262	2.5989
11	408.2897	0.0283	38	1497.4573	0.0835
12	423.7263	0.0565	39	1508.6812	3.0906
13	833.6008	54.4108	40	1517.3593	27.0056
14	846.4808	9.5193	41	2963.2883	15.8484
15	854.3177	23.0323	42	2963.6125	31.557
16	858.4449	2.1024	43	2975.5337	50.6693
17	892.4278	0.9477	44	2979.6203	0.2255
18	900.1121	6.0015	45	3084.1568	0.6266
19	949.1014	2.7433	46	3084.9036	3.5301
20	953.4699	0.8472	47	3087.291	1.3775
21	1125.7577	189.1564	48	3091.0951	1.9515
22	1131.2521	6.108	49	3131.2472	0.4031
23	1144.2706	23.7776	50	3131.9466	5.1509
24	1148.7234	1.4354	51	3141.527	0.8239
25	1211.6774	2.7981	52	3148.7571	1.1318
26	1219.0527	3.5888	53	3154.2429	10.7094
27	1371.1586	0.6993	54	3157.3404	6.1499

SVIII Transient ion signals and curve fitting coefficients

The transient ion signals from each isomer over the full measured range of -500 to 7500 fs are shown in Figures S4 through S8.

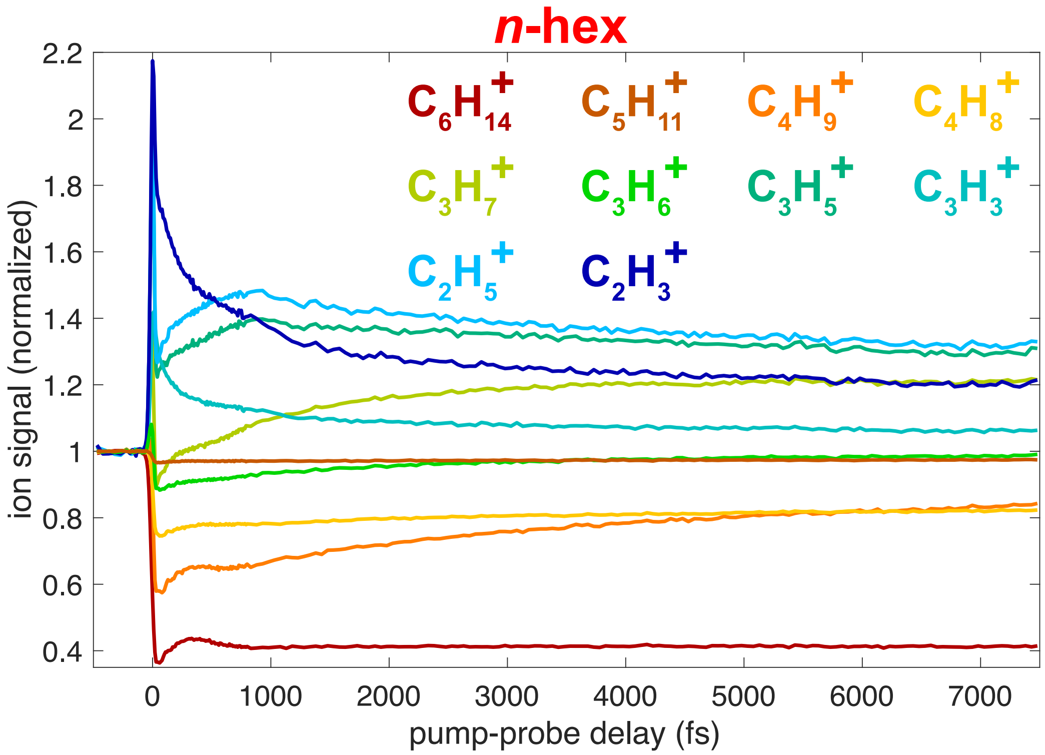

Figure S4: Transient ion signals for n-hexane.

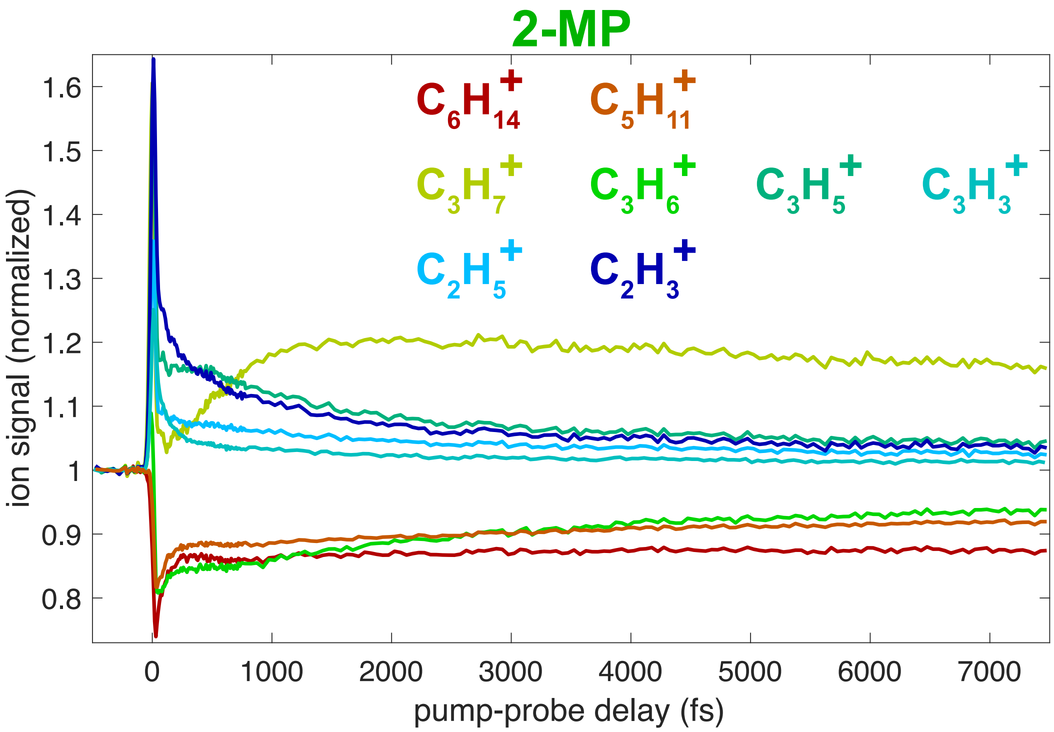

Figure S5: Transient ion signals for 2-methylpentane.

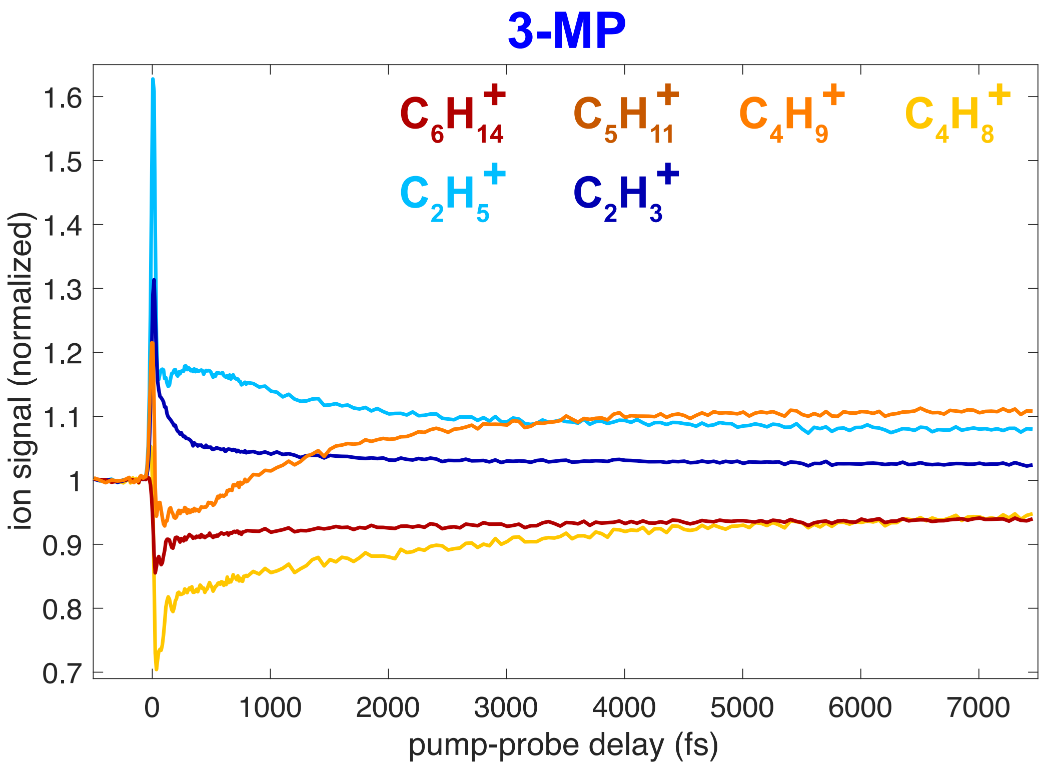

Figure S6: Transient ion signals for 3-methylpentane.

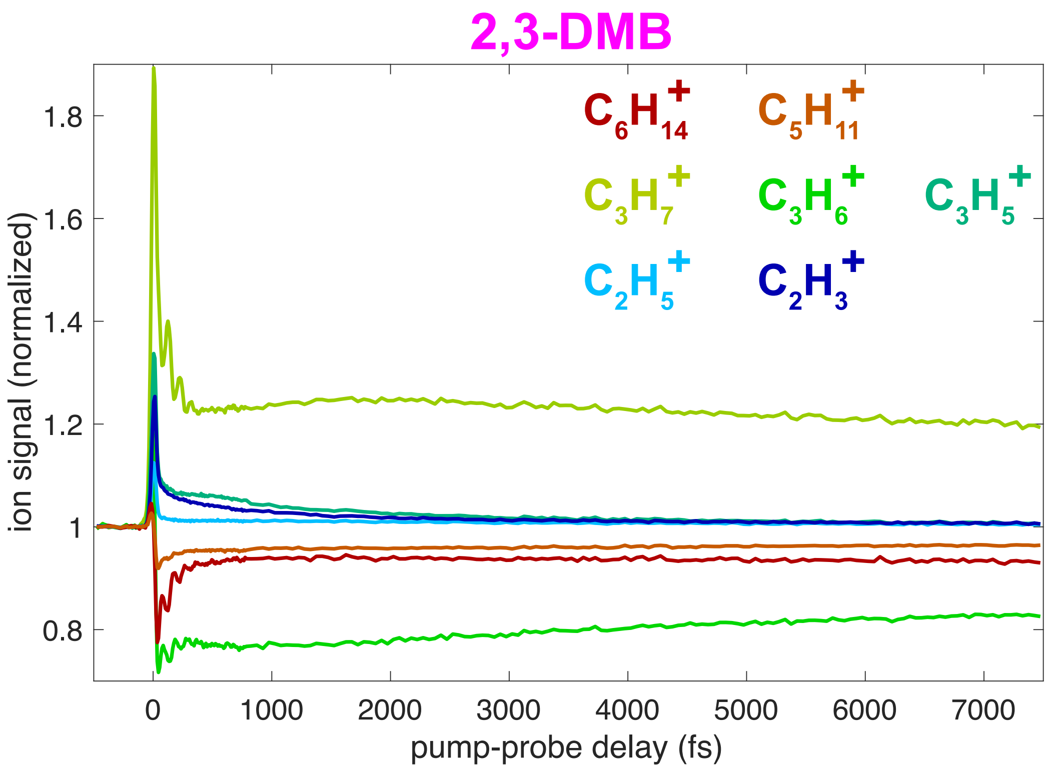

Figure S7: Transient ion signals for 2,3-dimethylbutane.

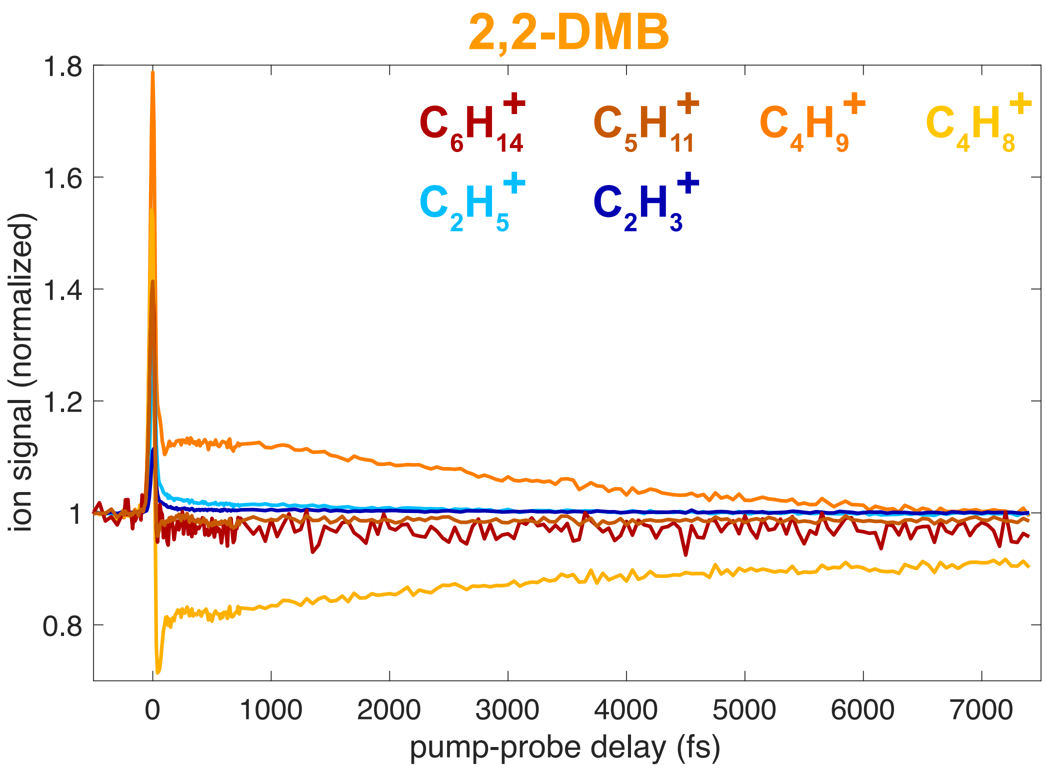

Figure S8: Transient ion signals for 2,2-dimethylbutane.

Where feasible, the transient ion signals as a function of pump-probe delay τ were fit to a series of exponential decay functions convoluted with the Gaussian IRF, following literature methods.[3] The overall fit equation consists of four terms:

$$S(\tau) = ae^{-\tau^2/s^2} + bP(\tau, T_1) + cP(\tau, T_2) + d(1 + \mathrm{erf}\left(\frac{\tau}{s}\right)) + 1 \tag{S.2}$$

where $s = 21$ fs was obtained from Figure S2. The constants a, b, c, d, T_1, and T_2 are variable parameters optimized by nonlinear least squares curve fitting in MATLAB.

The first term in eq S.2 simulates the IRF from the cross-correlation signal (Figure S2). The remaining terms in eq S.2 are associated with the dynamics of the transient ion signals due to excitation by the probe pulse. The terms $P(\tau, T_i)$, $i = 1, 2$ are given by

$$P(\tau, T_i) = \left[1 + \mathrm{erf}\left(\frac{\tau}{s} - \frac{s}{2T_i}\right)\right] e^{-\tau/T_i}, \tag{S.3}$$

and the last term in eq S.2 accounts for depletion or enhancement of an ion signal as $\tau \to \infty$ relative to its yield at $\tau < 0$.

The parent molecular ion (m/z 86) signals could be fit to eq S.2 for 2-MP, 3-MP, and 2,3-DMB, as shown in Figure S9. Fitting coefficients are given in Table S36.

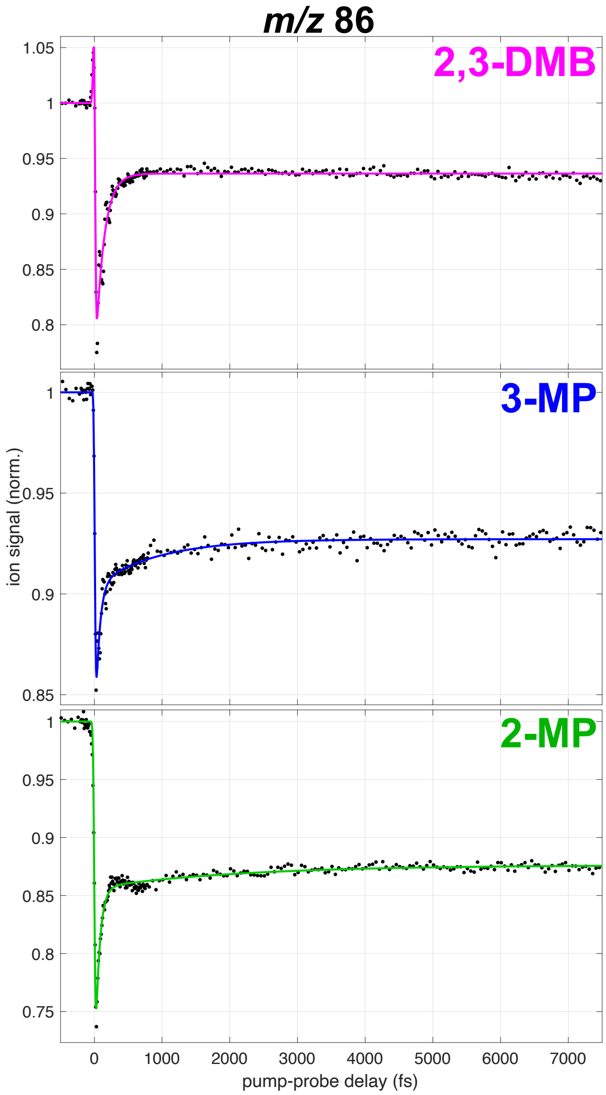

Figure S9: Fitted m/z 86 transient ion signals for 2-MP, 3-MP, and 2,3-DMB.

Table S36: Fit coefficients for parent molecular ions m/z 86.

Coeff.	2-MP	3-MP	23-DMB
a	-0.01±0.01	0.032±0.008	0.14±0.01
b	-0.089±0.008	-0.045±0.006	-0.094±0.005
T_1 (fs)	63±7	60±12	124±9
c	-0.098±0.008	-0.012±0.002	-
T_2 (ps)	2.1±0.9	0.9±0.2	-
d	-0.062±0.001	-0.0364±0.0004	-0.0318±0.0006

The $C_5H_{11}{}^+$ ion (m/z 71) signals could be fit to eq S.2 for n-hex, 2-MP, and 2,3-DMB, as shown in Figure S10. Fitting coefficients are given in Table S37.

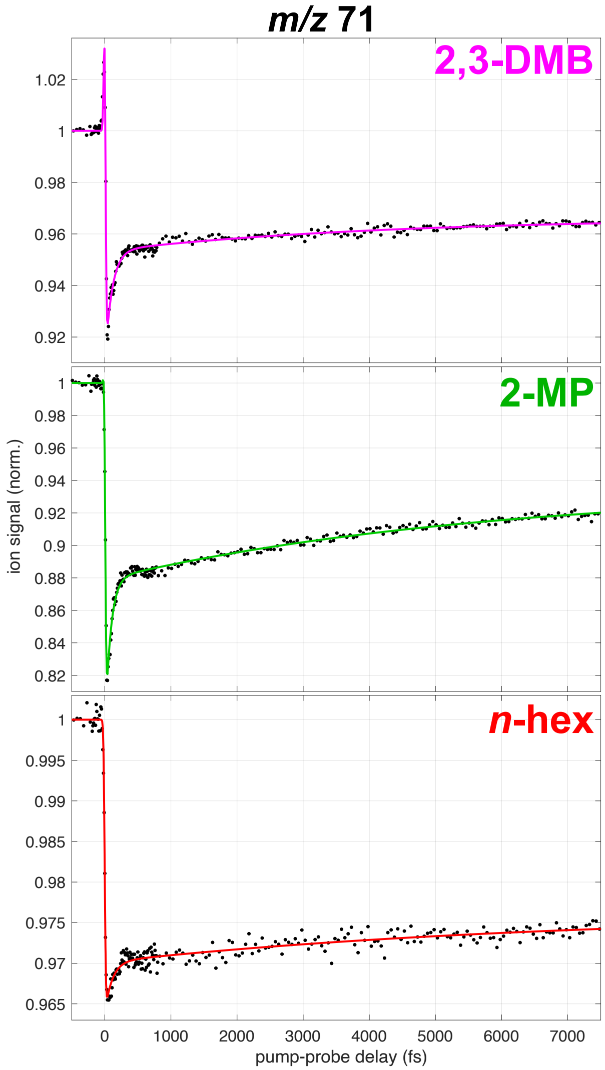

Figure S10: Fitted m/z 71 transient ion signals for n-hex, 2-MP, and 2,3-DMB.

Table S37: Fit coefficients for $C_5H_{11}^+$ ions m/z 71.

Coeff.	n-hex	2-MP	23-DMB
a	-0.002±0.001	0.049±0.005	0.068±0.004
b	-0.0031±0.0008	-0.050±0.003	-0.022±0.002
T_1 (fs)	110±40	81±8	103±13
c	-0.003±0.002	-0.012±0.002	-0.006±0.002
T_2 (ps)	>5	>5	4±2
d	-0.012±0.002	-0.032±0.004	-0.017±0.001

The $C_4H_9^+$ ion (m/z 57) and $C_4H_8^+$ (m/z 56) signals could be fit to eq S.2 for n-hex, 3-MP, and 2,2-DMB, as shown in Figure S11. Fitting coefficients are given in Table S38.

Table S38: Fit coefficients for $C_4H_9^+$ and $C_4H_8^+$ ions m/z 57 and 56.

Coeff.	n-hex (57)	n-hex (56)	3-MP (57)	3-MP (56)	22-DMB (57)	22-DMB (56)
a	0.06±0.01	0.003±0.005	0.268±0.0089	0.19±0.01	0.79±0.03	0.69±0.04
b				-0.109±0.009		
T_1 (fs)				66±7		
c	-0.127±0.004	-0.030±0.001	-0.073±0.001	-0.051±0.002	0.07±0.01	-0.065±0.006
T_2 (ps)	3.2±0.3	1.9±0.2	1.42±0.08	2.7±0.3	3.6±1.4	1.8±0.6
d	-0.071±0.004	-0.090±0.001	0.036±0.001	-0.038±0.002	0±0.01	-0.043±0.005

The $C_3H_7^+$ ion (m/z 43) and $C_3H_6^+$ (m/z 42) signals could be fit to eq S.2 for n-hex, 2-MP, and 2,3-DMB, as shown in Figure S12. Fitting coefficients are given in Table S39.

Table S39: Fit coefficients for $C_3H_7^+$ and $C_3H_6^+$ ions m/z 43 and 42.

Coeff.	n-hex (43)	n-hex (42)	2-MP (43)	2-MP (42)	23-DMB (43)	23-DMB (42)
a	0.37±0.02	0.114±0.007	0.65±0.02	0.179±0.009	0.64±0.03	0.35±0.02
b			-0.13±0.05	-0.029±0.008	0.17±0.02	-0.030±0.007
T_1 (fs)			800±300	60±20	83±15	60±40
c	-0.046±0.005	-0.049±0.001	0.07±0.04	-0.061±0.001	0.1±0.8	-0.1±0.1
T_2 (ps)	1.02±0.09	1.6±0.1	>5	2.9±0.3	>5	>5
d	0.104±0.003	-0.0075±0.0008	0.06±0.08	-0.027±0.002	0±0.8	0±0.1

The $C_3H_5^+$ ion (m/z 41) and $C_3H_3^+$ (m/z 39) signals could be fit to eq S.2 for n-hex, 2-MP, and 2,3-DMB (m/z 41 only), as shown in Figure S13. Fitting coefficients are given in Table S40.

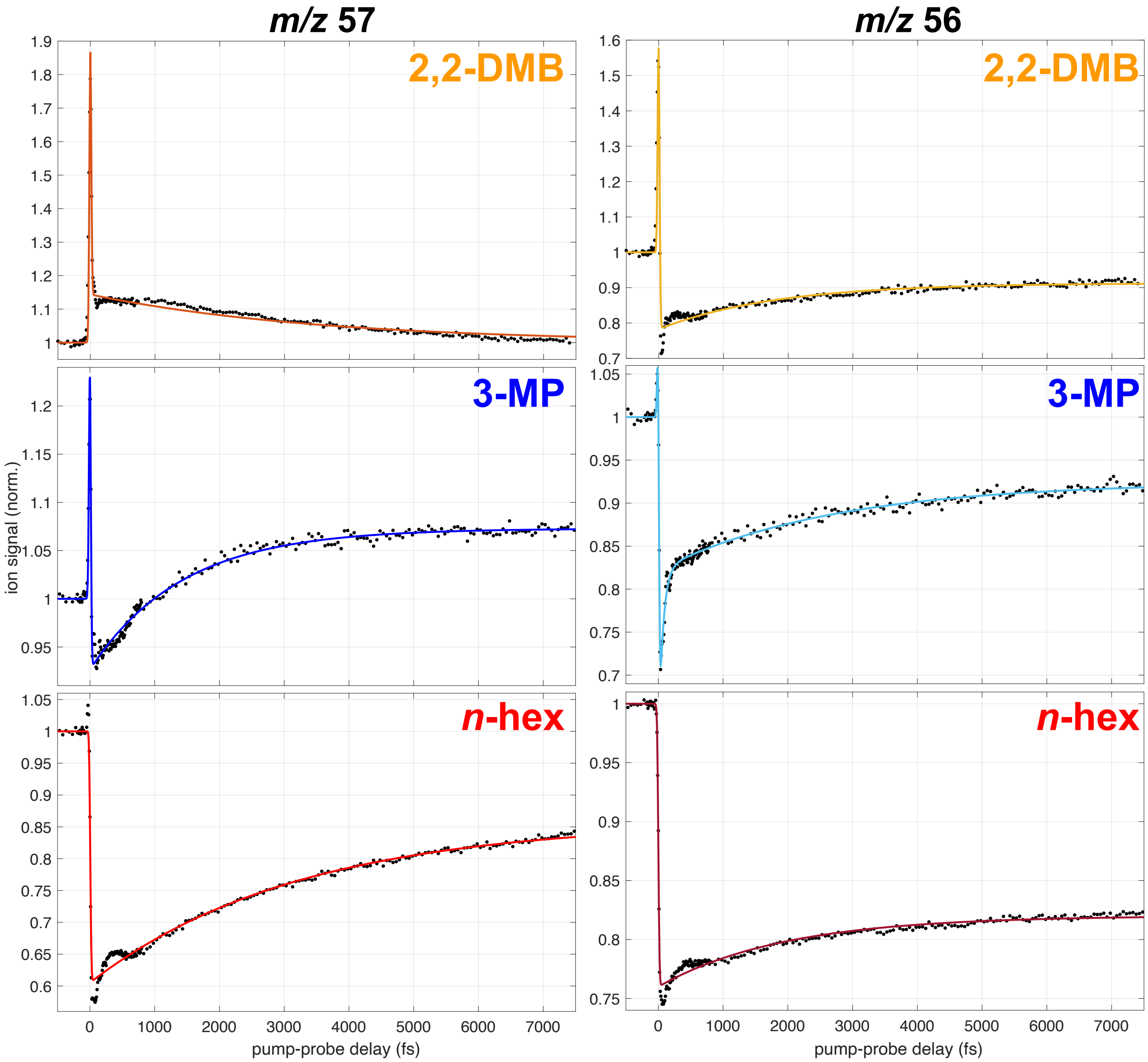

Figure S11: Fitted m/z 57 and 56 transient ion signals for n-hex, 3-MP, and 2,2-DMB.

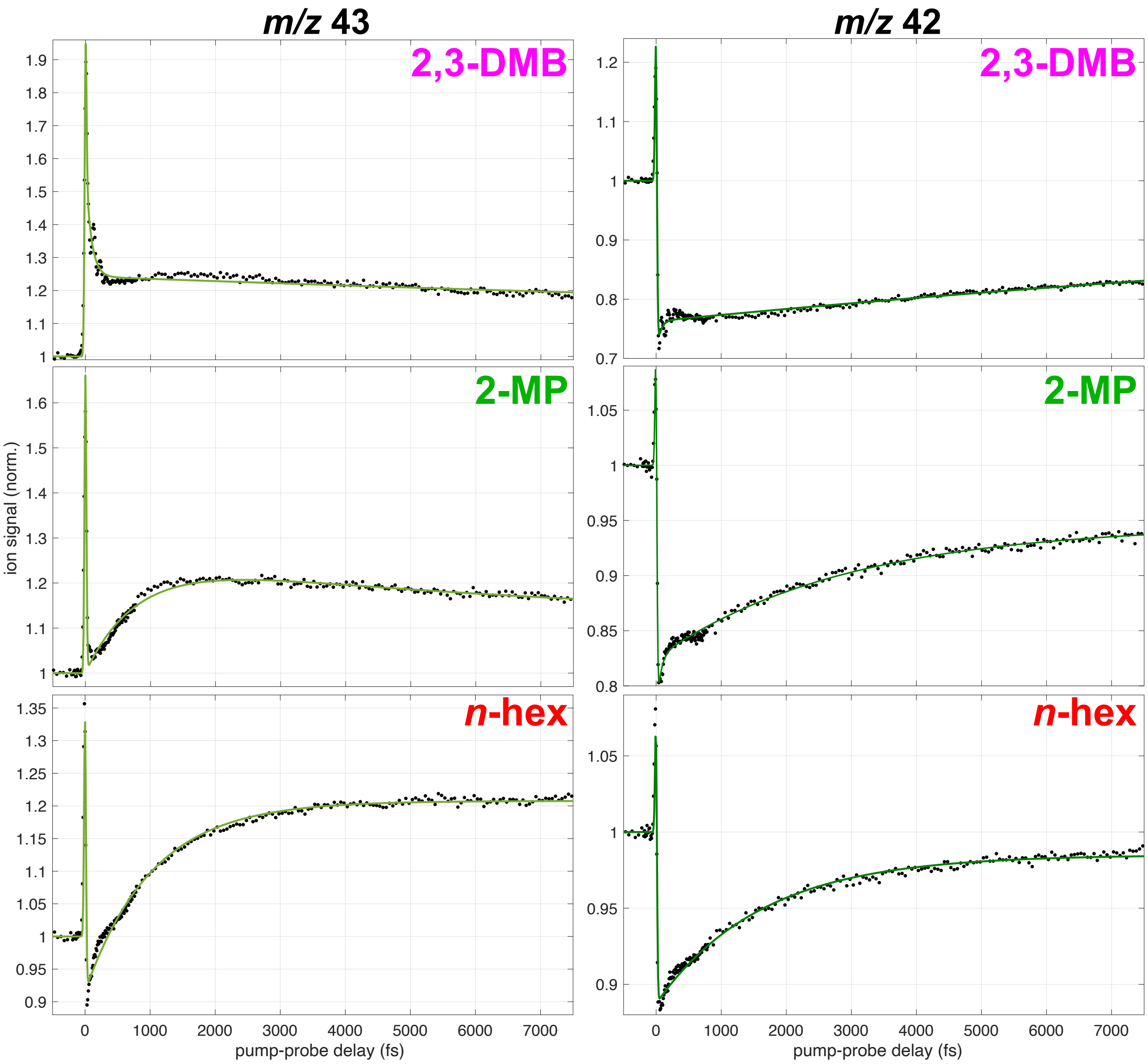

Figure S12: Fitted m/z 43 and 42 transient ion signals for n-hex, 2-MP, and 2,3-DMB.

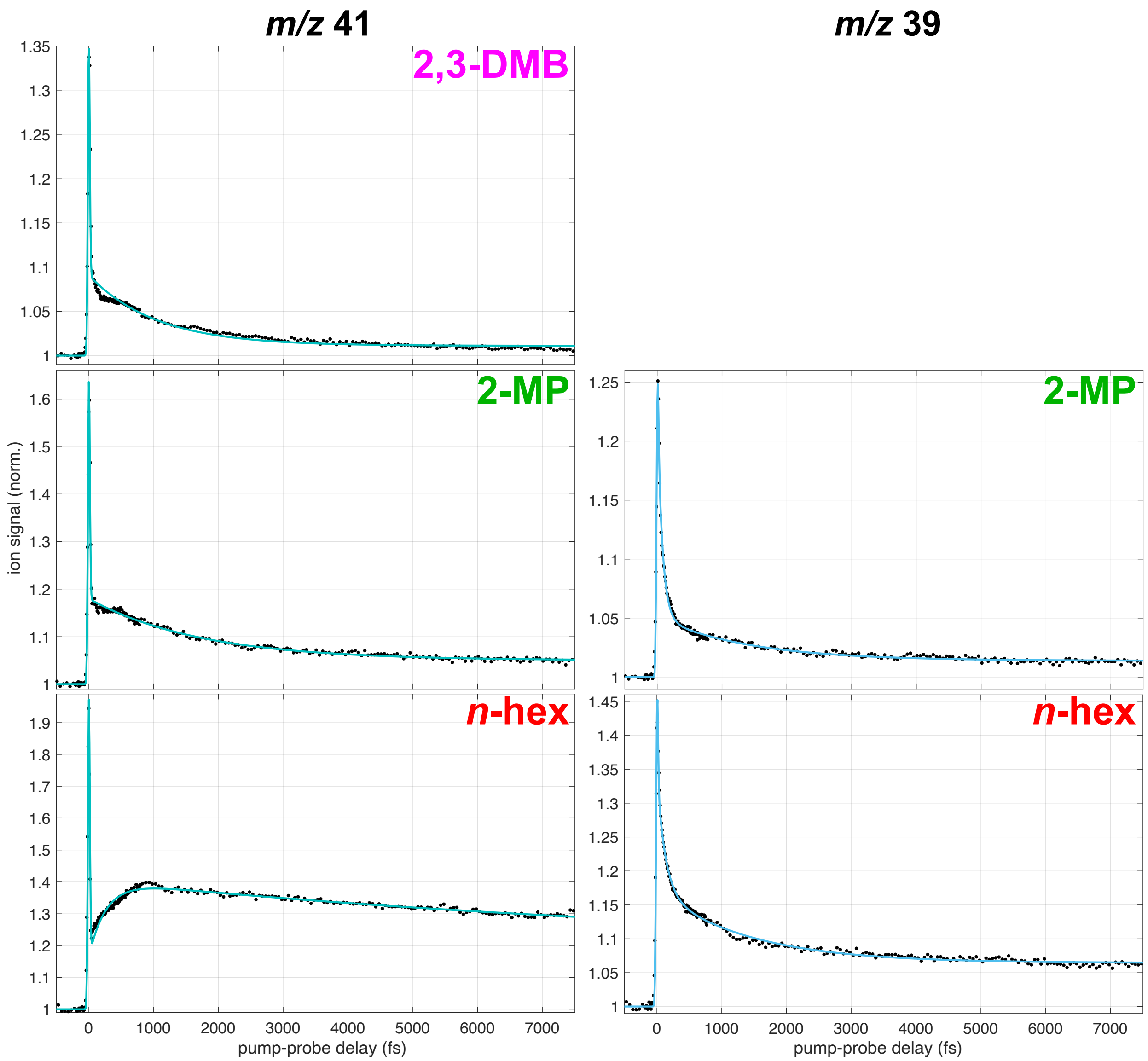

Figure S13: Fitted m/z 41 and 39 transient ion signals for n-hex, 2-MP, and 2,3-DMB.

Table S40: Fit coefficients for $C_3H_5^+$ and $C_3H_3^+$ ions m/z 41 and 39.

Coeff.	n-hex (41)	n-hex (39)	2-MP (41)	2-MP (39)	23-DMB (41)
a	0.88±0.03	0.26±0.01	0.54±0.01	0.121±0.006	0.30±0.01
b	-0.12±0.01	0.088±0.007		0.096±0.005	
T_1 (fs)	260±60	125±18		72±5	
c	0.1±0.1	0.053±0.004	0.065±0.002	0.019±0.002	0.040±0.002
T_2 (ps)	>5	1.4±0.2	1.7±0.2	1.4±0.3	1.0±0.1
d	0.1±0.1	0.032±0.001	0.025±0.002	0.0069±0.0006	0.006±0.001

The $C_2H_5^+$ ion (m/z 29) and $C_2H_3^+$ (m/z 27) signals could be fit to eq S.2 for *n*-hex, 2-MP, and 3-MP, as shown in Figure S14. Fitting coefficients are given in Table S41.

Table S41: Fit coefficients for $C_2H_5^+$ and $C_2H_3^+$ ions m/z 29 and 27.

Coeff.	n-hex (29)	n-hex (27)	2-MP (29)	2-MP (27)	3-MP (29)	3-MP (27)
a	0.90±0.03	0.82±0.03	0.33±0.01	0.39±0.02	0.51±0.02	0.15±0.01
b	-0.17±0.01	0.15±0.02		0.16±0.02		0.10±0.01
T_1 (fs)	180±30	280±80		50±8		77±8
c	0.11±0.02	0.14±0.03	0.031±0.002	0.081±0.005	0.056±0.003	0.020±0.003
T_2 (ps)	4±2	1.7±0.5	1.4±0.1	1.1±0.1	1.7±0.4	1.1±0.2
d	0.13±0.01	0.102±0.004	0.015±0.001	0.021±0.002	0.039±0.004	0.0135±0.0006

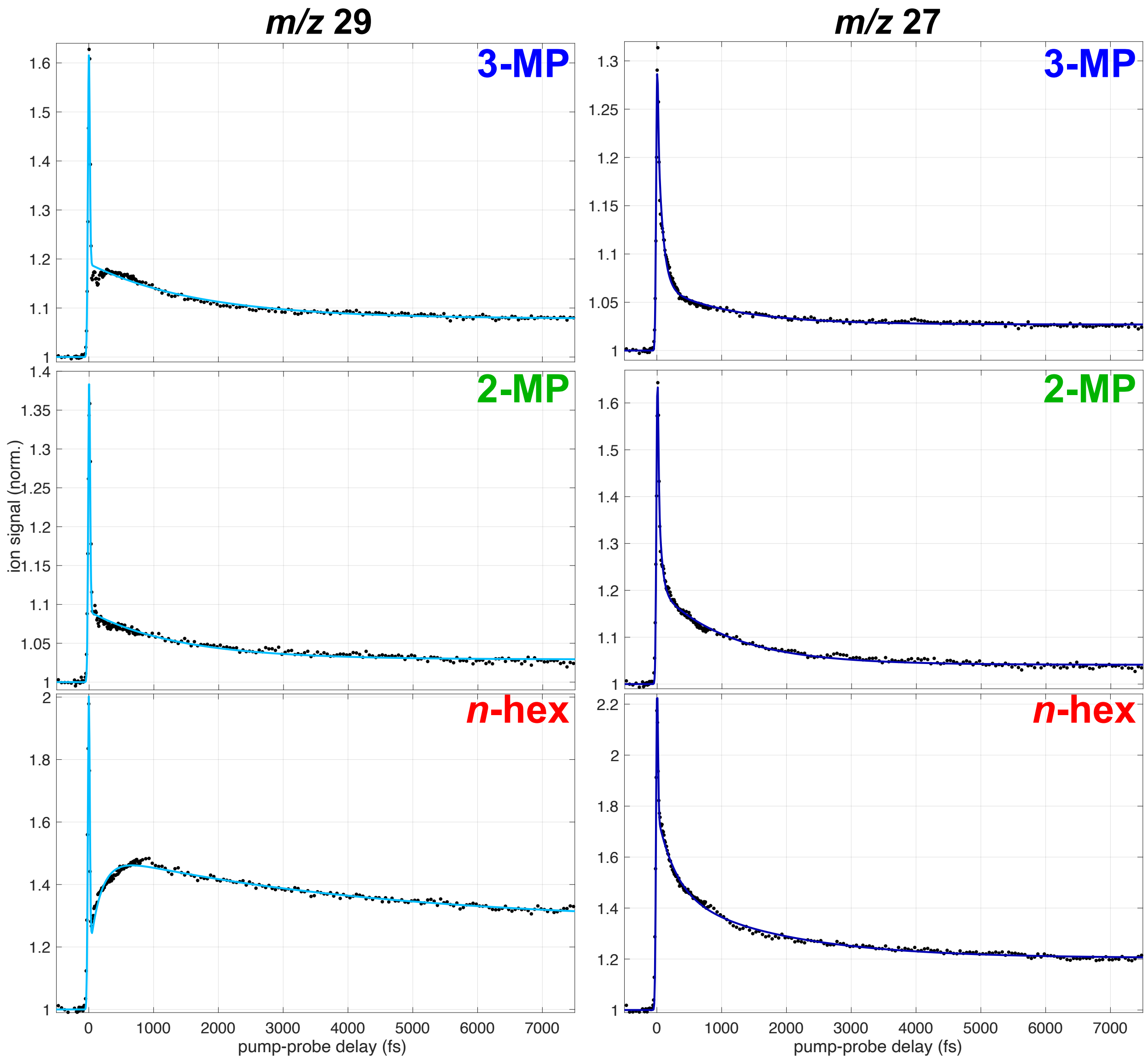

Figure S14: Fitted m/z 29 and 27 transient ion signals for n-hex, 2-MP, and 3-MP.

References

(1) Hankin, S. M.; Villeneuve, D. M.; Corkum, P. B.; Rayner, D. M. Intense-Field Laser Ionization Rates in Atoms and Molecules. *Phys. Rev. A* **2001**, *64*, 013405.

(2) Usachenko, V. I.; Pyak, P. E.; Kim, V. V. Comparative study of strong-field ionization in laser-irradiated F_2 and other diatomic molecules: Density-functional-theory-based molecular strong-field approximation. *Phys. Rev. A* **2009**, *79*, 023415.

(3) Jochim, B.; DeJesus, L.; Dantus, M. Ultrafast Disruptive Probing: Simultaneously Keeping Track of Tens of Reaction Pathways. *Rev. Sci. Instrum.* **2022**, *93*, 033003.
